# Age, sex, and mating status discrimination in the sand fly *Lutzomyia longipalpis* using near infra-red spectroscopy (NIRS)

**DOI:** 10.1186/s13071-023-06097-1

**Published:** 2024-01-12

**Authors:** Tainá Neves Ferreira, Lilha M. B. Santos, Vanessa Valladares, Catherine M. Flanley, Mary Ann McDowell, Gabriela A. Garcia, Clélia C. Mello-Silva, Rafael Maciel-de-Freitas, Fernando Ariel Genta

**Affiliations:** 1 Laboratório de Bioquímica e Fisiologia de Insetos, Instituto Oswaldo Cruz, Fiocruz, Rio de Janeiro, Brazil; 2 Laboratório de Transmissores de Hematozoários, Instituto Oswaldo Cruz, Fiocruz, Rio de Janeiro, Brazil; 3 Malacology Laboratory, Instituto Oswaldo Cruz, Fiocruz, Rio de Janeiro, Brazil; 4 Department of Biological Sciences, Eck Institute for Global Health, University of Notre Dame, Notre Dame, IN USA; 5 Instituto Nacional de Ciência e Tecnologia em Entomologia Molecular, Rio de Janeiro, Brazil

**Keywords:** *Lutzomyia longipalpis*, NIRS, Age, Discriminant classification, Sand fly, *Leishmania*

## Abstract

**Background:**

Understanding aspects related to the physiology and capacity of vectors is essential for effectively controlling vector-borne diseases. The sand fly *Lutzomyia longipalpis* has great importance in medical entomology for disseminating *Leishmania* parasites, the causative agent of Leishmaniasis, one of the main neglected diseases listed by the World Health Organization (WHO). In this respect, it is necessary to evaluate the transmission potential of this species and the success of vector control interventions. Near-infrared spectroscopy (NIRS) has been used to estimate the age of mosquitoes in different conditions (laboratory, semi-field, and conservation), taxonomic analysis, and infection detection. However, no studies are using NIRS for sand flies.

**Methods:**

In this study, we developed analytic models to estimate the age of *L. longipalpis* adults under laboratory conditions, identify their copulation state, and evaluate their gonotrophic cycle and diet.

**Results:**

Sand flies were classified with an accuracy of 58–82% in 3 age groups and 82–92% when separating them into young (<8 days) or old (>8 days) insects. The classification between mated and non-mated sandflies was 98–100% accurate, while the percentage of hits of females that had already passed the first gonotrophic cycle was only 59%.

**Conclusions:**

We consider the age and copula estimation results very promising, as they provide essential aspects of vector capacity assessment, which can be obtained quickly and at a lower cost with NIRS.

**Graphical Abstract:**

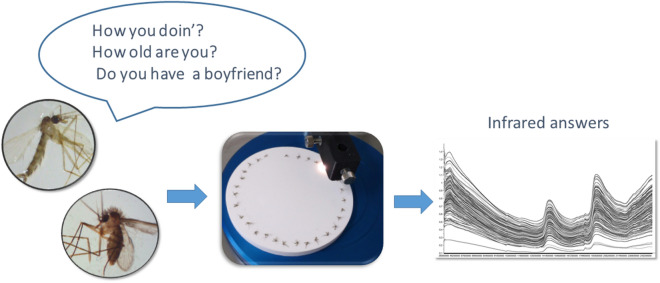

**Supplementary Information:**

The online version contains supplementary material available at 10.1186/s13071-023-06097-1.

## Key message

Determining adult dipterans sand flies' age and mating status may be necessary for critical assessment of vector control interventions. Near-infrared spectroscopy (NIRS) is a fast, low-cost technique with many advantages compared to the traditional techniques that follow ovarian or cuticle development or pteridine content. In this work, we show for the first time to our knowledge that NIRS may be used for discrimination between young and old sand flies and mated or non-mated adults of both sexes in the Leishmania vector *Lutzomyia longipalpis*.

**Supplementary Information:**

The online version contains supplementary material available at 10.1186/s13071-023-06097-1.

## Background

Leishmaniasis is a neglected disease and is considered one of the most important vector-borne diseases. Its complex transmission cycle presents a diversity of vectors, reservoirs, and parasites [40, 64]. It is found in almost all continents, apart from Australia and Antarctica. Leishmaniasis is present in approximately 90 countries in the tropics, subtropics, and southern Europe [8, 64]. It is estimated that around 350 million people are at risk of contracting the infection, particularly in places with low social and economic development [1, 8, 13, 42, 64].

Sand flies (family *Psychodidae*, subfamily *Phlebotominae*) are vectors of the parasites in the genus *Leishmania*, transmitted through the female bite during the blood meal. These hematophagous dipterans are distributed worldwide, particularly in tropical and sub-tropical regions [13, 40, 64].

There are several genera among phlebotomine sand flies, but *Lutzomyia* and *Phlebotomus* are the most significant in vector capacity [5, 40, 46]. In the Americas, *Lutzomyia longipalpis* is one of the most important species, being responsible for the development and transmission of *Leishmania infantum*, the parasite that causes visceral leishmaniasis [5, 6, 40].

In arthropod vector-borne diseases, the pathogens need time to develop and replicate inside the vector and reach their infective form. This time is called the extrinsic incubation period (EIP) [16]. Females of *L. longipalpis*, for example, need to live at least 7–9 days to transmit these parasites. They need to make a first infective blood-feeding, which can happen only 1–2 days after emergence, due to the time required for the development of the oral apparatus. Following that, the development of the metacyclic forms of *L. infantum* is completed at 6–7 days post-feeding. Then, the parasites may be transmitted in a second or multiple blood-feeding, closing the transmission cycle [28, 52]. The parameters EIP and longevity compose a mathematical model that evaluates the vectorial capacity of the insect [16].

In this context, knowledge about the age of the insects is essential to measure the impact of vector control strategies, such as chemical insecticides or attractive toxic sugar baits (ATSBs) interventions [22, 34]. For sand flies, this assessment is usually done by capture-mark-release [23]. However, this methodology primarily provides population density and may be influenced by environmental changes.

The classic techniques for determining the age of insects are the analyses of cuticular growth, ovarian development, and quantification of pteridines [2, 34, 63]. In the evaluation of ovaries, it is necessary to dissect each insect to infer whether a female has laid eggs (parous) or not (nulliparous) (Detinova 1962 [2]). Nevertheless, these techniques tend to be laborious, demanding time and specialized personnel to be carried out. These techniques are considered accurate and efficient. However, it has not been possible to analyze the necessary number of samples to evaluate an intervention’s effectiveness based on the sand fly populations’ age [26, 29, 35].

In recent times, alternative methods for age grading have been developed based on cuticular hydrocarbons [2], near-infrared reflectance spectroscopy (NIRS) [36, 54, 58, 59], and transcriptional profiles (gene biomarkers) [10, 11, 25]. In this sense, the use of near-infrared spectroscopy (NIRS) has been considered a viable and efficient alternative with high precision, fast results, and low cost to estimate the ages of insects of different orders [4, 33, 37, 47, 54].

Near-infrared spectroscopy provides information on the interaction of radiation with matter (atoms and molecules). When infrared radiation is absorbed, it produces vibrational transitions depending on the molecules present in the sample, particularly of biological origin. This information creates spectral profiles according to the internal and external biochemical composition of the organism [2, 9, 36, 60].

The cuticles of insects are composed of organic compounds such as esters, ketones, alcohols, sterols, and mainly hydrocarbons [01]. These molecules form a lipid layer to protect insects from dehydration and abrasion. Importantly, these biological materials containing bonds such as –CH, –OH, –SH, and –NH present rotation, elongation, and bending that are detected by light in the range from 350 to 2500 nm [4, 21, 43, 48].

In this respect, NIRS can detect physiological changes in the insect and has been successfully used in different arthropods, with diverse aims such as determining cryptic species, infection, type of diet, or physiological state [14, 31, 36, 49, 53, 54, 58, 59, 61].

This technique also had good results in different applications in vectors species like mosquitoes [17, 33, 37, 49, 54, 55, 58, 59], kissing bugs [14, 61], culicoids [47], snails [62], and in agricultural pests [27, 50]. Nevertheless, there are no studies with leishmaniasis vectors. In the last years, studies have used NIRS analysis to correctly classify young (< 7 days) and old (> 7 days) *Anopheles* spp. [54] with high accuracy (78–89% and 73.5–97%, respectively). In other work, the same authors also obtained good results for the classification of young (< 7 days) and old (> 7 days) colonized mosquitoes through cross-validation analysis [32] or in *Aedes aegypti* wild-type and *Wolbachia* infected [58, 59]. Liebman et al. [35] verified whether varied diets would influence age prediction using NIRS, and they had 71–90% of correct predictions classifying *A. aegypti* into young vs. old females. Recently, [37] showed better results in predicting whether wild mosquitoes were parous or nulliparous, using an autoencoder and neural networks, which represents an improvement of this technique.

Thus, in this work, we investigated NIRS in several aspects related to the physiology of sand flies, age estimative, copula state, gonotrophic cycle, and diet, to increase the information about NIRS as an effective tool to evaluate interventions for the control of these insect vectors.

## Material and methods

### Sand flies rearing

All sand flies, *L. longipalpis*, used in this study were reared in laboratory conditions (temperature of 26 ºC ± 2 ºC and relative humidity of ≥ 70%), and this population originally came from Jacobina, Bahia, Brazil (11°10′54″ S; 40°30′43″ W). After they emerged from the pupae, the adults were kept in cubic tissue cages with approximately 25 cm on each side. Adult sand flies were fed with 70% (w/v) autoclaved sucrose solution in cotton wool for routine rearing. For blood-feeding and oviposition, females were fed on golden hamsters (*Mesocricetus auratus*) anesthetized with xylazine (10 mg/kg) plus ketamine (200 mg/kg) or fed on rabbit blood (*Oryctolagus cuniculus*) in an artificial system (Hemotek) using chick skin as a membrane [12, 19, 38].

### Near-infrared spectrophotometry scanning

Sand flies were scanned immediately after being anesthetized and killed with ethyl acetate. They were placed on a plate (spectralon) to be scanned individually. Each insect was positioned laterally under the NIR probe, taking the head and thorax region and scanning an area of around 2 mm. This process was done with a Labspec 4i NIRS spectrometer (Malvern Panalytical, Longmont, CO) containing an external 3.2-mm-diameter fiber optic probe and an 18.6 W light source (Model 135325 Rev B, ASD Inc.), according to previous works published [36, 49]. The ASD software RS^3^ version 3.1 was used to collect all spectra, taking around 5 s per sample collected. One single spectrum was collected per sample, changing the plate position slightly until we observed the best resolution. Spectra were collected in the range of 350 to 2500 nm, with a resolution of 1 nm.

**Fig. 1:**
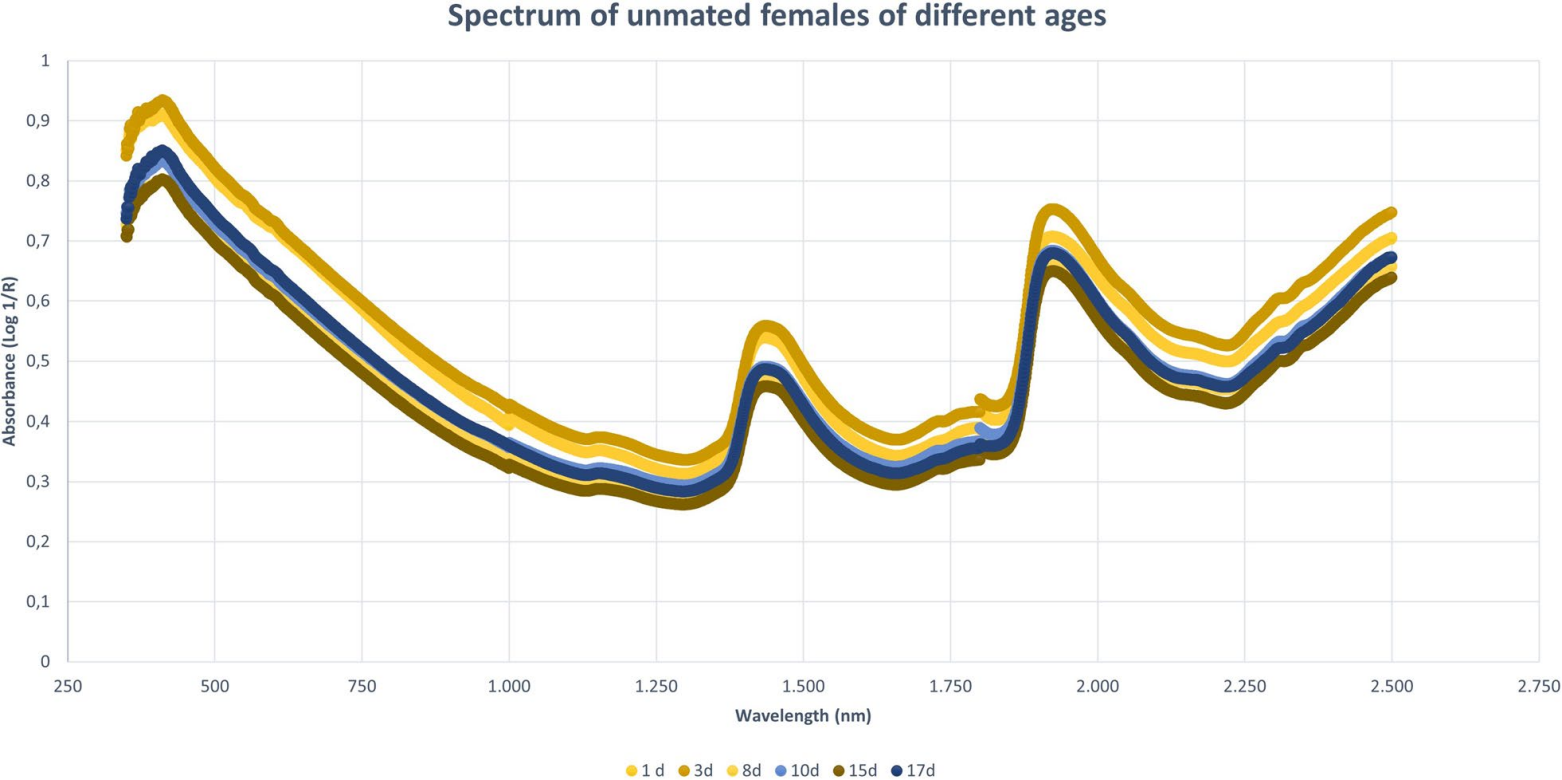
Examples of spectra for sand flies (adult *Lutzomyia longipalpis* females): absorbance (Log 1/R) vs. wavelength (nanometers)

In Fig. 1, we can see typical spectra Log 1/R (absorbance) vs. wavelength (nm) obtained for adults of *L. longipalpis*.

According to the experimental scheme, all insects were previously fed with sucrose solution and anesthetized for spectra collection on different days post-emergence.

## Samples

### Longevity analysis

Adult sand flies, newly emerging (0–1 day) males and females, were confined in different cubic tissue cages. These Insects were removed to scan in the NIR probe on different days post-emergence: 1 (0–1 day old), 3, 8, 10, 15, and 17. These sand flies were kept separate and did not have the opportunity to copulate, and this protocol was repeated four times to obtain at least 10 spectra for each age and sex (range 11–92). This analysis was repeated by taking young (1 and 3 days), middle (8 and 10 days), and old (15 and 17) sand flies together. A new classification was done considering only two groups: young sand flies < 8d (1 and 3 days) vs. ≥ 8d (8, 10, 15, and 17 days). Table 1 presents the number of sand flies scanned and used in calibration and classification.

**Table 1:** Classification of the hits of sand flies (*Lutzomyia longipalpis*) of different ages separated by gender, based on a linear discriminant analysis (LDA)

A	Females				Males			
Age (days)	Model (*n*)	Validation (*n*)	Hits	Accuracy (LDA)	Model (*n*)	Validation (*n*)	Hits	Accuracy (LDA)
1	20	41	76%	62%	27	17	73%	50%
3	20	7	29%		40	52	56%	
8	20	13	100%		20	9	22%	
10	20	19	21%		20	14	50%	
15	20	14	7%		8	4	40%	
17	6	5	60%		10	5	60%	
Total	106	99			125	101		

B	Females				Males			
Age (days)	Model (*n*)	Validation (*n*)	Hits	Accuracy (LDA)	Model (*n*)	Validation (*n*)	Hits	Accuracy (LDA)
1–3	40	48	85%	73%	67	69	80%	58%
8–10	40	32	78%		40	23	17%	
15–17	26	19	42%		18	9	44%	
Total	106	99			125	101		

C	Females				Males			
Age (days)	Model (*n*)	Validation (*n*)	Hits	Accuracy (LDA)	Model (*n*)	Validation (*n*)	Hits	Accuracy (LDA)
< 8	40	49	83%	87%	68	75	87%	86%
≥ 8	65	51	85%		47	36	92%	
Total	105	100			115	111		

A similar procedure was made with adult sand flies that newly emerged; however, this time, both sexes were kept in the same cage and had the chance to copulate. Posterior analyses such as ovary analysis to confirm whether females were fertilized were not made because of the sensibility of sand flies. During the scanning process, insects were carefully manipulated using blunt forceps, but we still had cases of loss of legs or other injuries in their bodies. Furthermore, the time between insects being anesthetized and scanned caused post-mortem dehydration, preventing subsequent dissection. In this assay, insects were scanned 1, 8, and 15 days post-emergence, and this procedure was done in triplicate. Table 2 (part A) presents the number of sand flies scanned and used in calibration and classification. In a second classification, we consider only young sand flies < 8d (1 day) vs. > 8d (15 days) (Table 2-part B).

**Table 2:** Classification of the hits of sand flies (*Lutzomyia longipalpis*) at different ages maintained together, based on a linear discriminant analysis (LDA)

A	Females				Males			
Age (days)	Model (*n*)	Validation (*n*)	Hits	Accuracy (LDA)	Model (*n*)	Validation (*n*)	Hits	Accuracy (LDA)
1	50	62	80%	75%	50	34	74%	82%
8	50	91	58%		60	48	67%	
15	50	54	87%		34	30	87%	
Total	150	207			144	112		

B	Females				Males			
Age (days)	Model (*n*)	Validation (*n*)	Hits	Accuracy (LDA)	Model (*n*)	Validation (*n*)	Hits	Accuracy (LDA)
< 8	50	62	97%	91%	40	44	80%	92%
> 8	45	59	93%		35	29	93%	
Total	95	121			75	73		

### Copulation status

The samples described in the item “Longevity analysis” were analyzed comparatively to verify the similarity between sand flies coupled and not copulated. For this, we analyzed samples of males and females only 8 days post-emergence, and the spectra were the same as obtained in the previous description. Table 3 shows the number of sandflies used in the two analyses following the same procedure described previously.

**Table 3:** Classification of the hits of sand flies adults (*Lutzomyia longipalpis*), copulated or not, based on a linear discriminant analysis (LDA)

A	Females				Males			
Status	Model (*n*)	Validation (*n*)	Hits	Accuracy	Model (*n*)	Validation (*n*)	Hits	Accuracy
NC	20	13	92%	100%	20	9	100%	100%
CP	20	25	100%		20	24	100%	
Total	40	38			40	33		

B	Females				Males			
Status	Model (*n*)	Validation (*n*)	Hits	Accuracy	Model (*n*)	Validation (*n*)	Hits	Accuracy
NC	22	11	82%	99%	23	12	100%	98%
CP	45	44	98%		40	44	100%	
Total	67	55			63	56		

*NC* not copulated and *CP* copulated

### Gonotrophic cycle

Sand flies were confined in two different cubic tissue cages with 85–100 females newly emerged (0–1 day) and fed with sucrose solution. Three days later, a group of females was fed with blood in an artificial system (item “Insects”), and 2–3 days after that, all engorged females were put in plastic pots with a plaster layer for oviposition. After 3 days, when these females completed 9–10 days post-emergence and after laying their eggs, they were anesthetized and scanned in the NIR wavelength region. This experiment was done in triplicate.

For the construction of the model, we scanned only females without visible eggs in their ovaries. This procedure simulated a field situation where it is possible to catch females without the clear observation of a blood feed or eggs in development. In this way, we tested whether NIRS can classify these females considering only the changes in their bodies before and after oviposition, without the interference from eggs. Those females that still had unlaid eggs were used only for prediction (classification). Table 4 presents the number of females employed in the analysis, highlighting that the second analysis (right part) was done by removing the females with eggs not laid independently of their rank.

**Table 4:** Classification of the hits of sand flies females (*Lutzomyia longipalpis*) fed on sugar solution or sugar plus blood based on a linear discriminant analysis (LDA)

	Females			A	Females			B
Status	Model (*n*)	Validation (*n*)	Hits	Accuracy	Model (*n*)	Validation (*n*)	Hits	Accuracy
Suc	60	143	43%	61%	60	143	43%	61%
Suc_B	60	150	59%		60	87	59%	
Total	120	293			120	230		

*Suc* Sucrose solution and *Suc_B* sucrose solution plus blood fed

### Chemometric analyses

The chemometric analyses were made in the Unscrambler R software (version 10.5.1) according to Valladares et al. [62] and Shang et al. [51]. Each spectrum was pre-processed to decrease possible noise (Smoothing Savitzky-Golay filter—2nd polynomial order/31 points and 1st derivative Savitzky-Golay—2nd polynomial order/15 points) to minimize the potential influence on results. A specific spectral model was created for each cluster of samples before their validation. Data for calibration and validation were chosen randomly. For model construction, we chose a similar “n” for each replica, totaling at least 40 samples, when possible. The remaining spectra were used in validation. These numbers are described in Tables 1, 2, 3, and 4. Principal component analysis (PCA) was the exploratory method used to validate smaples' belonging to distinct groups that corresponded to the different conditions (age, copula, and gonotrophic cycle) (Additional file 1: Figs. S1, S4, S7, S8, S9, and S10). Samples classified as outliers were identified and removed using Hotelling T^2^ statistics, considering a 95% confidence interval ellipse according to previous studies [39], and then a new PCA was recalculated without outliers.

**Fig. 2:**
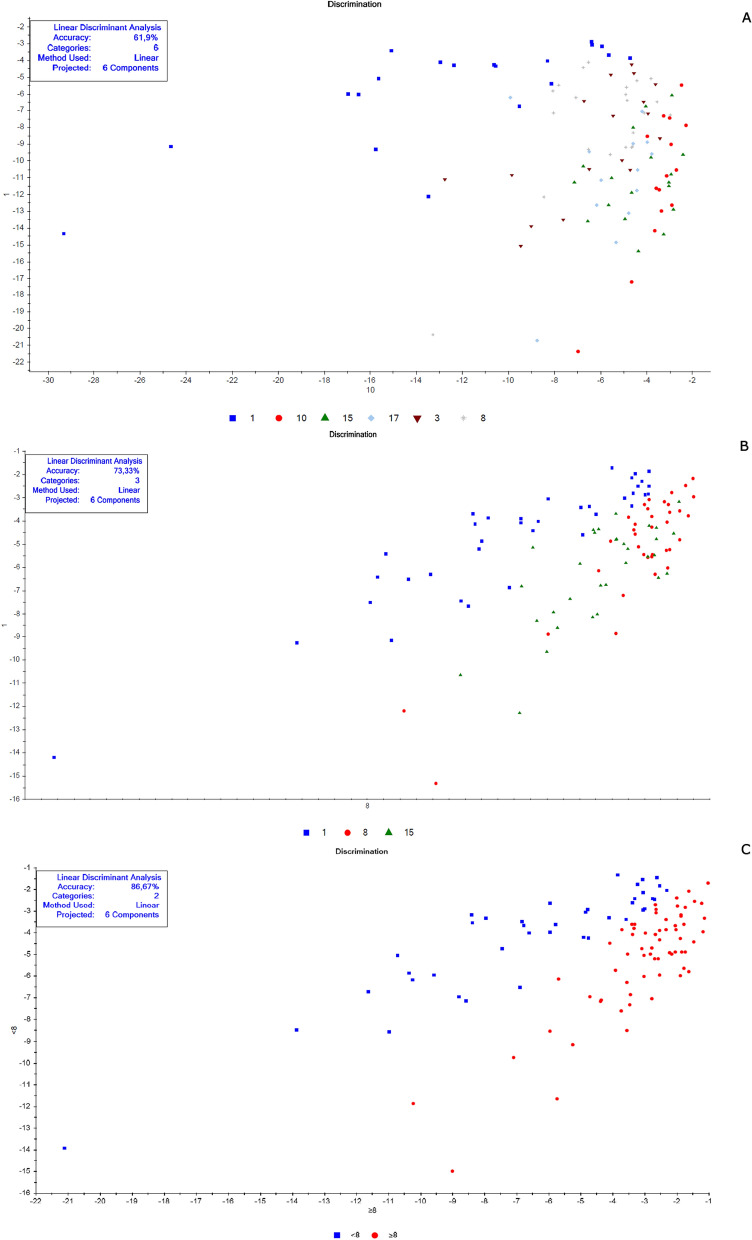
Linear discriminant analysis (LDA) of non-copulated *Lutzomyia longipalpis* females at different ages. **a** One (blue squares), 3 (brown triangles), 8 (gray stars), 10 (red circles), 15 (green triangles), and 17 (light blue diamonds) days old; **b** 1 (blue squares), 8 (red circles), and 15 (green triangles) days old; **c** < 8 (blue squares) or ≥ 8 (red circles) days old

The next step was classifying the results using a linear discriminant analysis (LDA). This tool linearly describes the main characteristics through the categorized information, ordering the samples in a particular group. The results are summarized in tables containing the number of spectra used in the validation, classification, the percentage of correct classifications (hits) and the accuracy of the LDA (Figs. 2, 5, 8, 9, 11, and 13).

**Fig. 3:**
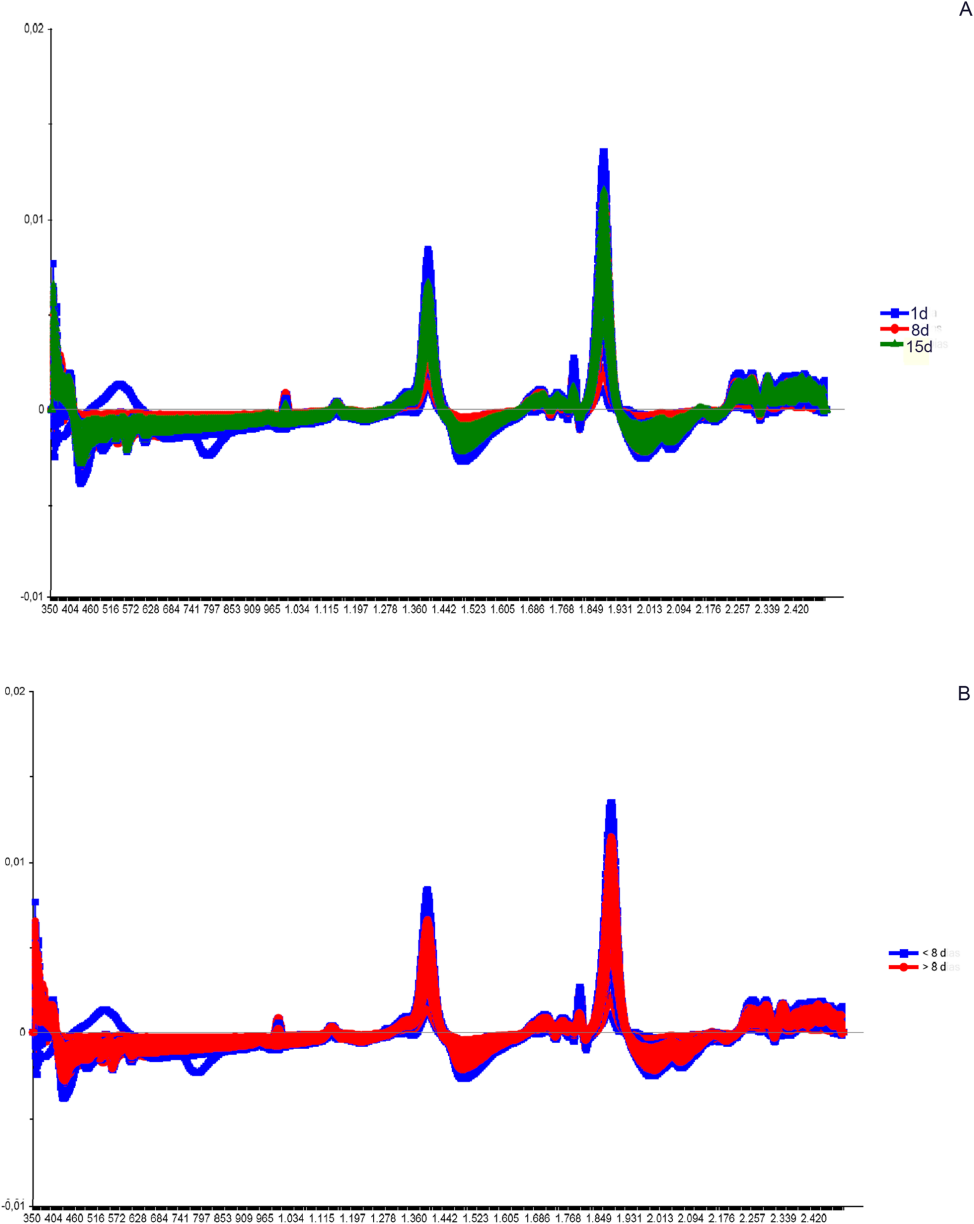
Spectra of non-copulated *Lutzomyia longipalpis* females at different ages (absorbance vs. wavelength—nm) after smoothing and first derivative pretreatment by Savitzky-Golay filter. **a** One (1, 8, and 15 days old; **b** < 8 or ≥ 8 days old

**Fig. 4:**
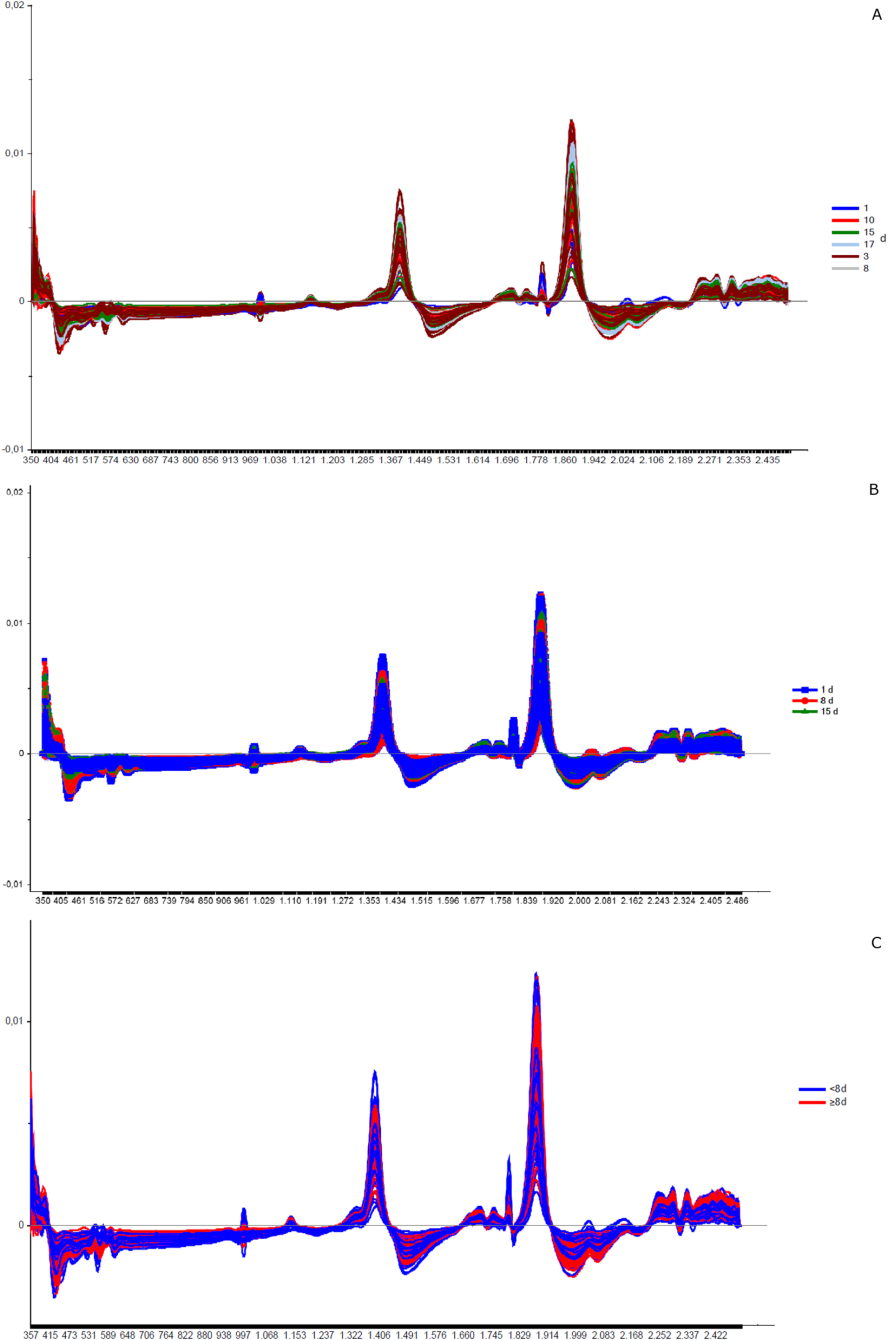
Spectra of non-copulated *Lutzomyia longipalpis* males at different ages (absorbance vs. wavelength, nm) after smoothing and first derivative pretreatment by Savitzky-Golay filter. **a** 1, 3, 8, 10, 15, and 17 days old; **b** 1, 8, and 15 days old; **c** < 8 or ≥ 8 days old

**Fig. 5:**
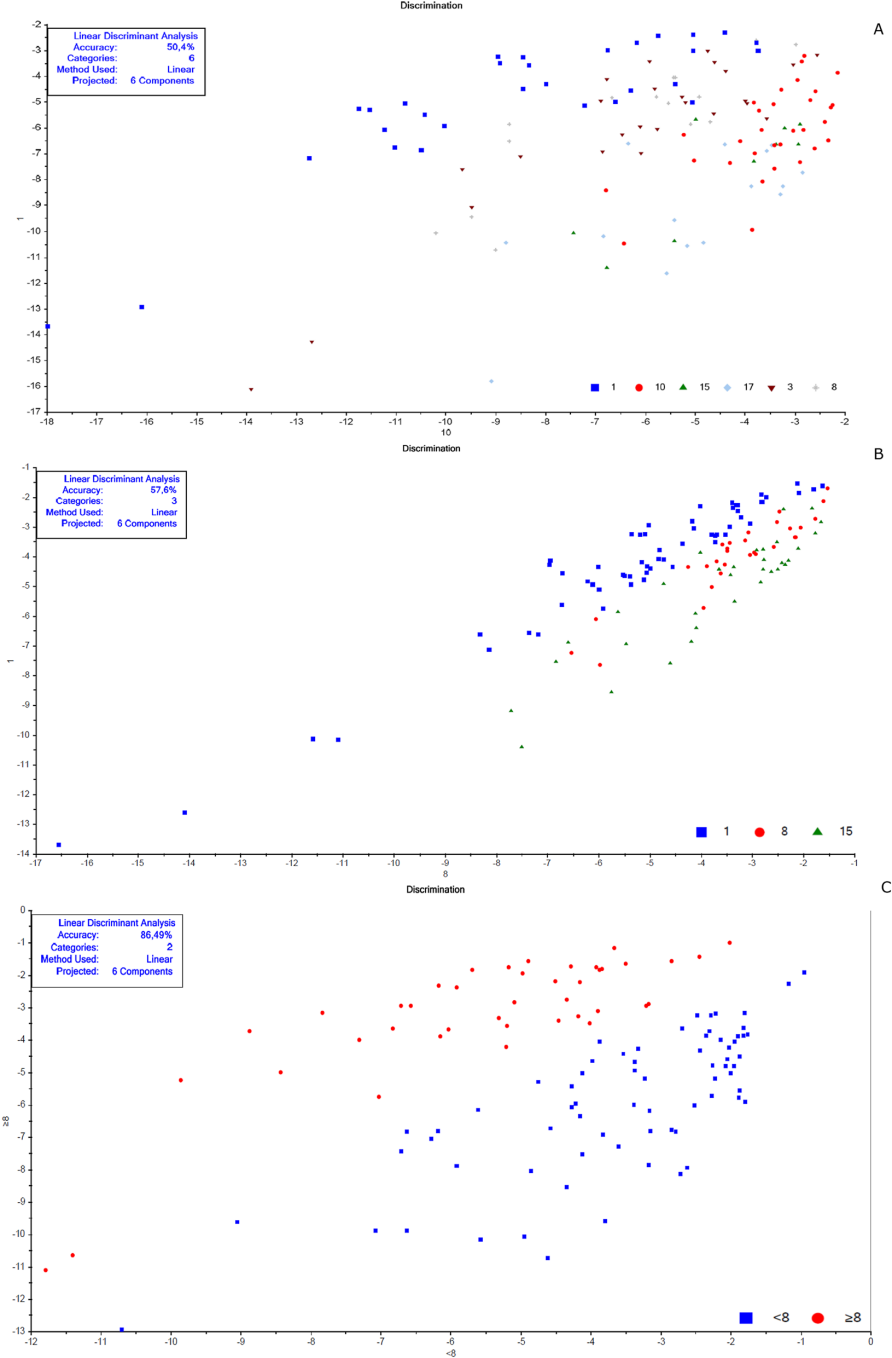
Linear discriminant analysis (LDA) of non-copulated *Lutzomyia longipalpis* males at different ages. **a** One (blue squares), 3 (brown triangles), 8 (gray stars), 10 (red circles), 15 (green triangles), and 17 (light blue diamonds) days old; **b** 1 (blue squares), 8 (red circles), and 15 (green triangles) days old; **c** < 8 (blue squares) or ≥ 8 (red circles) days old

**Fig. 6:**
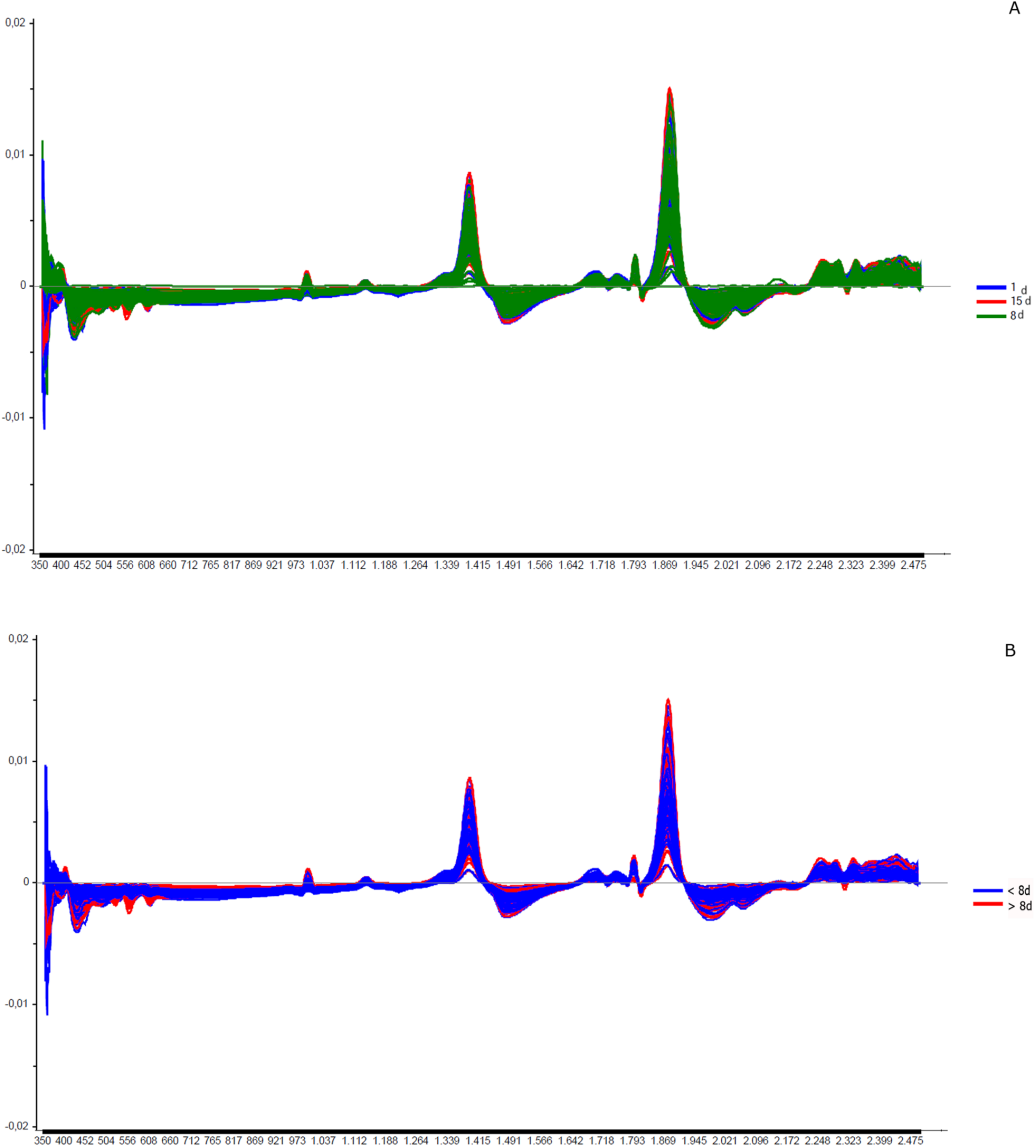
Spectra of copulated *Lutzomyia longipalpis* females at different ages (absorbance vs. wavelength, nm) after smoothing and first derivative pre-treatment by Savitzky-Golay filter. **a** 1, 8, and 15 days old; **b** < 8 or > 8 days old

**Fig. 7:**
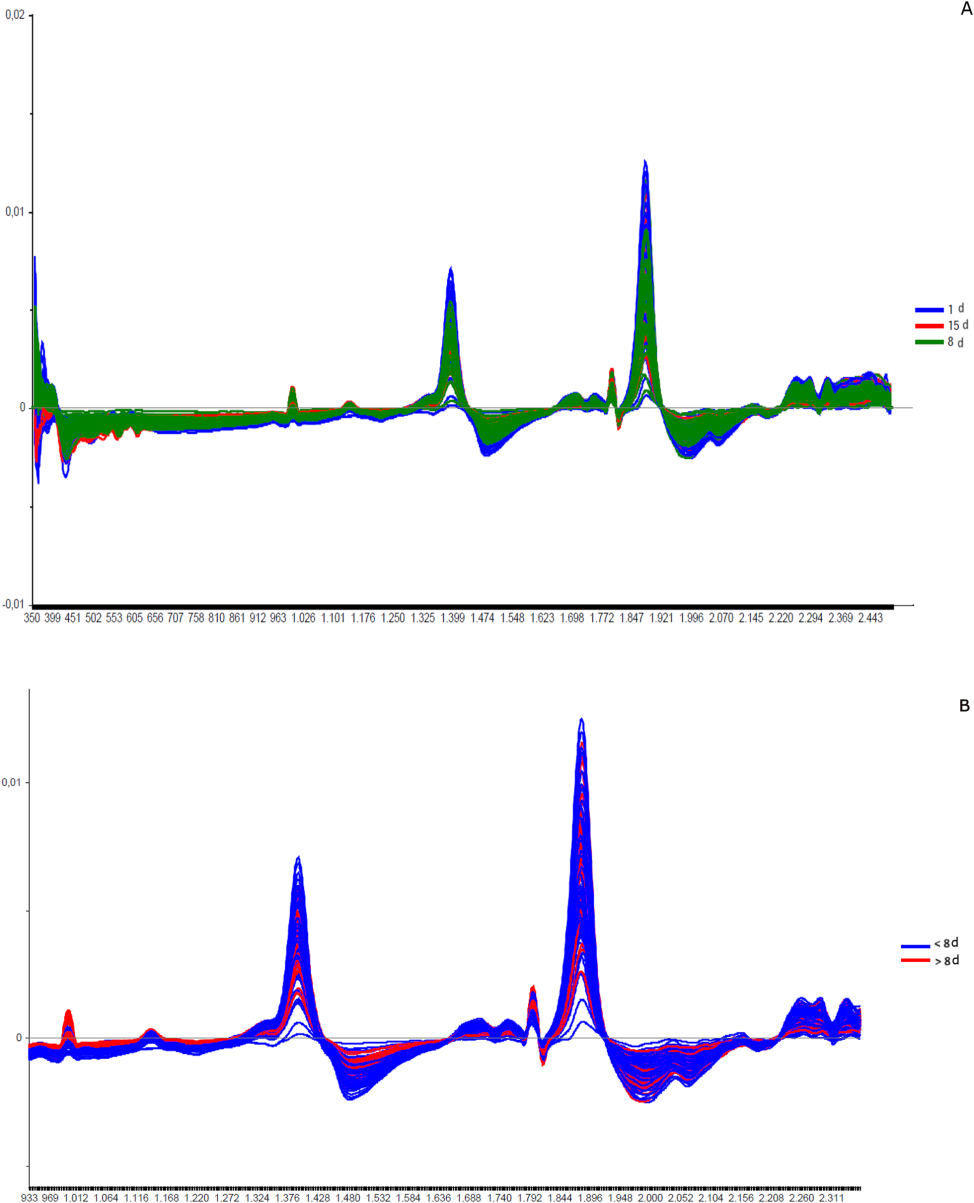
Spectra of copulated *Lutzomyia longipalpis* males at different ages (absorbance vs. wavelength) after smoothing and first derivative pre-treatment by Savitzky-Golay filter. **a** 1, 8, and 15 days old; **b** < 8 or > 8 days old

**Fig. 8:**
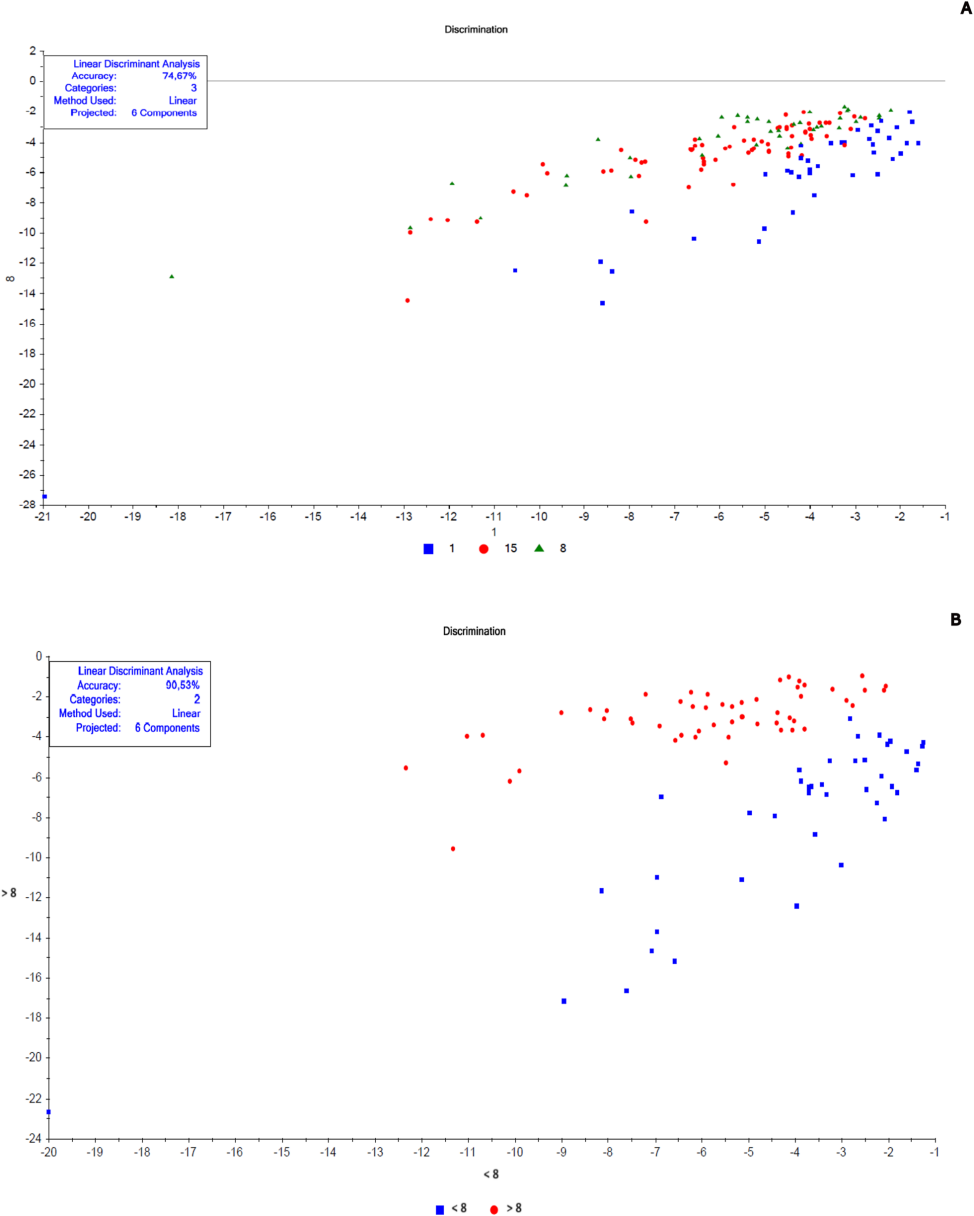
Linear discriminant analysis (LDA) of copulated *Lutzomyia longipalpis* females at different ages. **a** 1 (blue squares), 8 (green triangles), and15 (red circles) days old and **b** < 8 (blue squares) or > 8 (red circles) days old

**Fig. 9:**
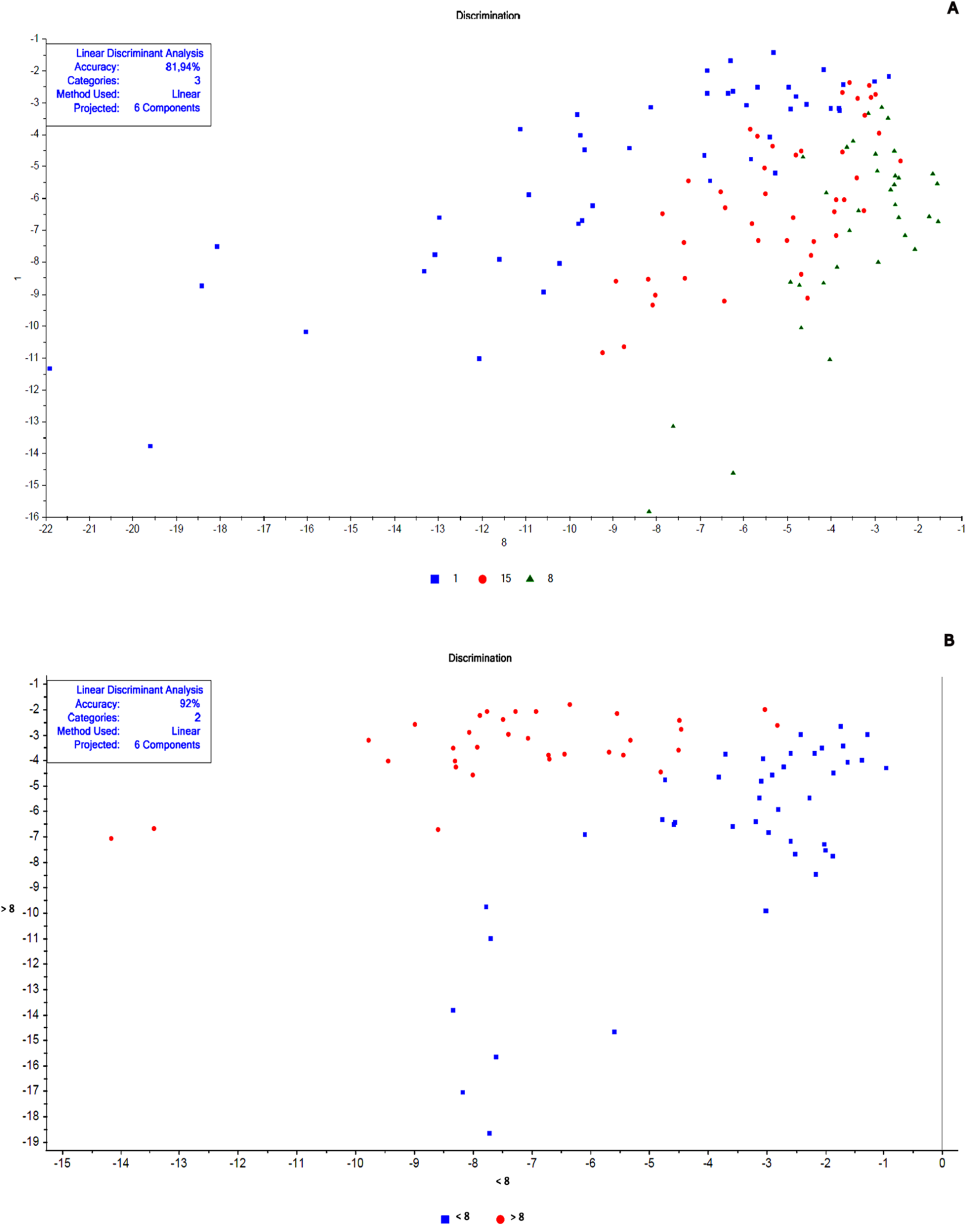
Linear discriminant analysis (LDA) of copulated *Lutzomyia longipalpis* males at different ages. **a** One (blue squares), 8 (green triangles), and15 (red circles) days old and **b** < 8 (blue squares) or > 8 (red circles) days old

**Fig. 10:**
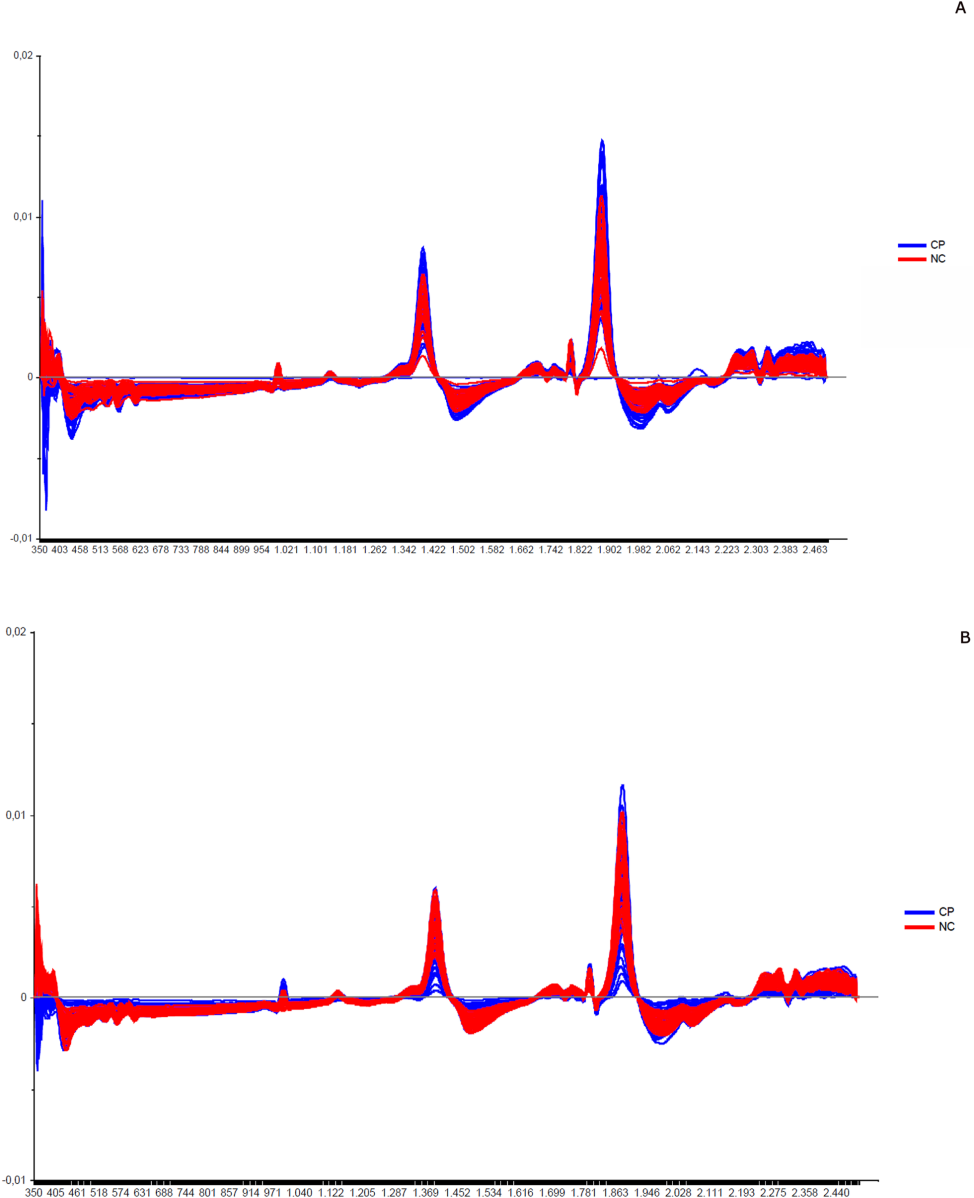
Spectra of copulated (CP) vs. not copulated (NC) of *Lutzomyia longipalpis* in same age (absorbance vs. wavelength, nm) after smoothing and first derivative pre-treatment by Savitzky-Golay filter. **a** females; **b** males

## Results

This work explored NIRS in different parameters of sand flies’ physiology: age, copulation, and gonotrophic cycle. Figure 1 presents the average spectra of females collected at six different time points: 1, 3, 8, 10, 15, and 17 days after emergence. These spectra show us that the main peaks were close to 1400, 1800, and 1900 nm. The PCA results for these females showed a clear separation between the youngest ones, especially at 1 day of age, and the others (> 8 days old) that overlapped in the upper quadrants (Additional file 1: Figure S1a). This was reflected in the linear discriminant analysis (LDA), which had an accuracy of only 62% (Fig. 2a), and in the correct age prediction (hits), which was < 60% for females with 3, 10, and 15 days (Table 1a).

The spectral profile for non-mated males was similar to the data observed in females, presenting the same peaks even after data processing with smoothing and Savitzky-Golay derivative (Fig. 4a). These peaks can be observed in the loading figures obtained by PCA (Additional file 1: Figure S3a).

The PCA data from males were relatively clustered, and it was impossible to determine the separation of samples by age (Additional file 1: Figure S4a). For females, accuracy was also low, only 50% (Fig. 5a), and hits were > 60% only for males 1 and 17 days old (Table 1a). Generally, females presented better scores than males (Table 1a; Fig. 2A and 5).

When insects were separated as young, middle, and old (Fig. 3a—females; Fig. 4b—males), the main peaks remained around 1400 and 1900 nm (Additional file 1: Figure S2a and Additional file 1: Figure S3b). The peaks from younger samples appear to have slightly greater amplitude compared to those from older insects (Fig. 3a—females, Fig. 4b—males). There was an improvement in the accuracy in the age classification of the females (Table 1b and Fig. 2b) and in the hits with a hit rate ≥ 78% for young and middle females. Sample clustering was readily observed in younger samples (1–3 days old) by PCA (Additional file 1: Figure S1b) and LDA (Fig. 2b).

In males, the accuracy did not presented a significant improvement (Table 1b and Fig. 5b). The correct rating was also low, 44 and 42%, for 15–17 days for males and females, respectively (Table 1b).

Grouping the insects into two classes, < 8 or ≥ 8 days (young or old), resulted in correct predictions > 83% (Table 1c), and this categorization could also be observed in the PCA for females and males (Additional file 1: Figure S1c and S4c). In this condition, the accuracy was > 85% for both sexes (Fig. 2C and 5C). No changes were detected in the main peaks for females (Fig. 3b and Additional file 1: Figure S2b) or males (Fig. 4c and Additional file 1: Figure S3c). Table 2 and Figs. 6, 7, 8, and 9 present the classification results at different ages of sand flies that were kept together in the same cage so they had the opportunity to mate. Figure 6a presents the main discriminant peaks for young, middle, and old females, which were around 1400 and 1900 nm, with no visible changes in amplitudes between samples, even after preprocessing or loadings obtained by PCA (Additional file 1: Figure S5a). The same peaks were observed for females separated as young and old, < 8 or > 8 days old (Fig. 6b and Additional file 1: Figure S5b). This profile was maintained for samples from males classified as young, middle, and old, or in < 8 or > 8 day olds (Fig. 7 and Additional file 1: Figure S6).

Regarding the principal component analysis, there was no formation of groups in samples for females or males divided into young, middle, and old (Additional file 1: Figure S7a, S8a), except for 15-day-old males. This aspect improved when samples were separated into young insects (< 8 days old) vs. old (> 8 days old) (Additional file 1: Figure S7b, Figure S8b).

In LDA results, except for the 8-day-old insects, the other ages had correct predictions > 74% (Table 2a). The accuracy of LDA was 75% for females and 82% for males (Table 2a, Figs. 8a and 9a).

By taking out the middle-aged sand flies and keeping only young vs. old, we improved the correct classification results, which were between 80 and 97% (Table 2b). The separation of the groups could be perceived in PCA analyses (Additional file 1: Figs. S7b and S8b), as well as accuracy obtained, > 91% (Table 2b; Figs. 8b and 9b).

To verify whether we could combine the spectra of mated and unmated insects in the age estimation, we compared these samples to each other at the same age (8 days) (Table 3). We detected a slight increase in amplitude in the main peaks of copulated females compared to non-copulated females (Fig. 10).

**Fig. 11:**
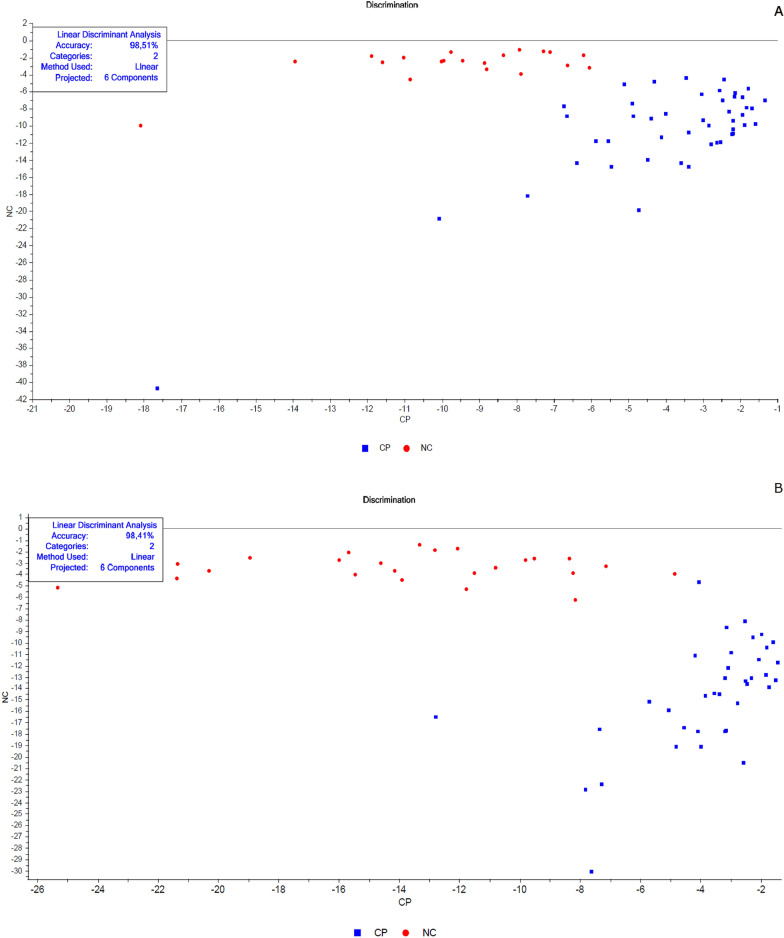
Linear discriminant analysis (LDA) of copulated (CP, blue squares) vs. not copulated (NC, red circles) **a** females and **b** males

PCA and LDA analyses were repeated, increasing the number of samples (Table 3). The result was very similar (Table 3) with accuracy > 98% and correct previsions among 82–100%. PCA (Additional file 1: Figure S9) and LDA (Fig. 11a—females and b—males) showed a clear partition between samples using less (Table 3a) or more samples (Table 3b, Additional file 1: Figure S9, and Fig. 11).

**Fig. 12:**
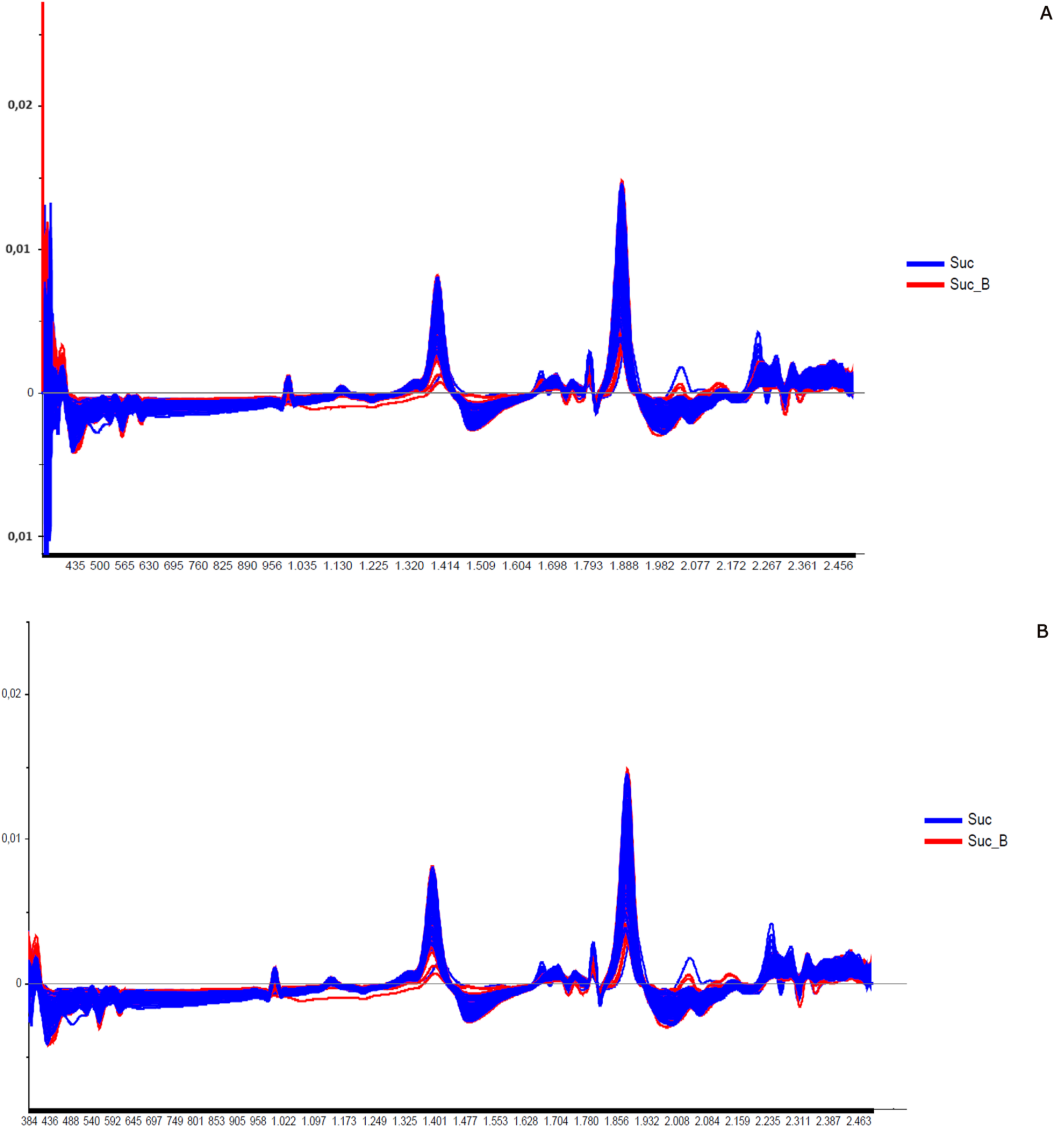
Spectra of females fed on sucrose solution or sucrose plus blood (absorbance vs. wavelength, nm) after smoothing and first derivative pre-treatment by Savitzky-Golay filter. (**a**) Results keeping females with unlaid eggs and (**b**) results without females with unlaid eggs

Another aspect related to the physiology of sand flies investigated was the gonotrophic cycle. We tested whether NIRS can categorize females fed only with sucrose or sucrose solution and blood after laying eggs. We saw that the discriminant peaks remain at 1400 nm and 1900 nm, with no bases broadening or changes in peak heights. However, we noticed the presence of more small peaks in the range of 1700–1780 and 2200–2300 nm, even after smoothing and derivative preprocessing (Fig. 12) or taking out samples from females with unlaid eggs.

**Fig. 13:**
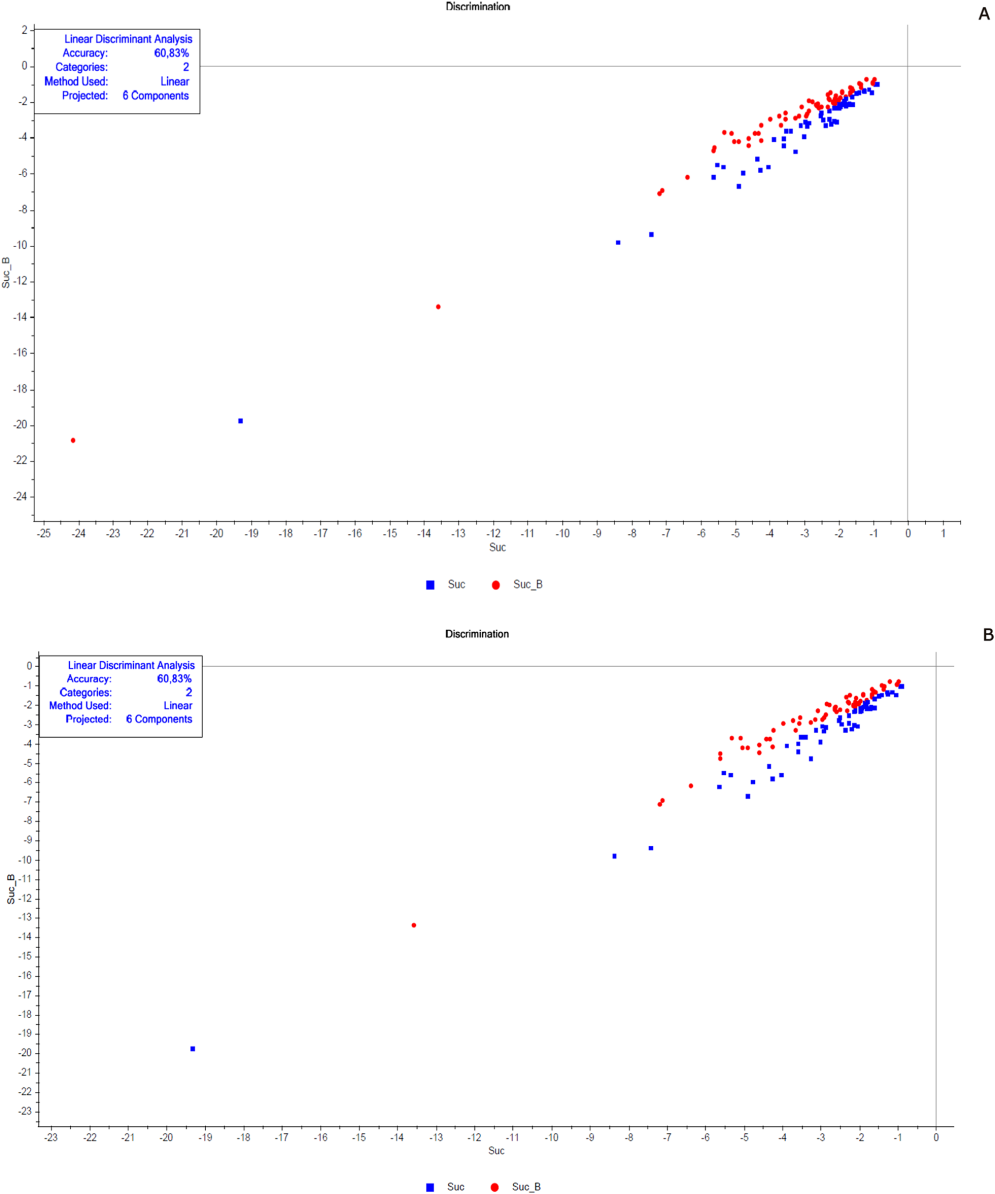
Linear discriminant analysis (LDA) of females fed on sucrose solution (Suc, blue squares) or sucrose plus blood (Suc_B, red circles). **a** Results keeping females with unlaid eggs and **b** results without females with unlaid eggs

PCA presented an overlap between samples from females that fed on sugar alone or sugar plus blood (Additional file 1: Figure S10). The correct classification for the sucrose group was only 43%, while for sucrose plus blood it was 59% (Table 4). The same result in hits and accuracy (61%) was found when taking out females with eggs unlaid (fewer samples) in predictions (Table 4b). In Fig. 13 we saw a mixture of the different samples in LDA.

## Discussion

The results presented in this work suggest that NIR spectroscopy could discriminate sand flies of different ages, mate status, and gonotrophic cycle for females, with varying accuracy depending on the condition tested. This tool has been used to estimate the age of other dipterans [4, 49, 54, 58, 59] but has not been applied before to leishmaniasis vectors.

The spectra collected demonstrated differences in chemical phenotypes that are putatively related to the metabolic arrangement observed in the diverse physiological conditions tested. In general, the main peaks observed at 1400–1450, 1780–1800, and 1900–1930 nm are associated with peaks of water absorption (Fig. 1), with a profile like that observed in mosquitoes [18, 49]. In studies with other insects such as mosquitoes and beetles [3, 20, 44], the peak at 1820 nm is attributed to the first overtone of C–H stretching and the peak at 1900 nm corresponds to the absorption of COOC functional groups, which is due to the lipid composition of the cuticle of these insects. These functional groups are according to the main functions found in cuticles, hydrocarbons, and fatty acids [01, 51].

Younger sand flies seem to have a higher body water content than older ones, demonstrating that water could influence age grading in insects [36, 39]. This was observed when we scanned sand flies kept on silica for > 7 days, since the spectra lose this characteristic shape, staying without or with lower peaks (data not shown), and also in Figs. 1, 3, 4B and C, 6, and 7, for example, where we see a tendency of reducing height of the peaks for samples of sand flies > 8 days old.

One of the main aims of this study was to estimate the age of sand flies, which is an essential topic in vector control. These data make it possible to assess impacts on the transmission cycle since the pathogens have an incubation period in the vector before reaching their infective forms [32, 36]. Furthermore, by estimating the age structure of medically relevant vectors, researchers can infer a given population’s ability to transmit a pathogen. Females of *L. longipalpis*, for example, need to complete at least 7–9 days to be able to develop the metacyclic forms of *Leishmania* spp. and disseminate them in a second blood feeding [02, 28, 52].

The results of age grading using insects separated by sex and six different ages had a low ratio of correct predictions, but this was improved when we split the samples into three (young, middle, old) or two (young or old) groups, with correct predictions > 83%. Similar results were found in the literature with *Anopheles* spp. [36, 54], *Aedes* spp. [58, 59], culicoides [47], and *Drosophila* spp. [4].

Previous works also had problems with correctly classifying ages between 10 and 16 days old, similar to our results for 10- and 15-day-old females and males (Table 1a). This suggests that the functional groups measured decreased with the age of the sand flies [35]. It is possible that biochemical and structural changes in insects, detectable by NIRS, only occur when age differences are ≥ 7 days [36], which agrees with the findings for the flesh fly, *Sarcophaga bullata*, in the analysis of cuticular HC composition [2].

Notably, sandflies are more fragile than mosquitoes, and it is difficult to collect them in high numbers because of mortality in older insects. This was especially critical for getting flies older than 10 days since their average life span is 15 days [19]. However, if we consider 8 days as the limit to assess the vector's capacity to spread *Leishmania*, the low ability of NIRS to discriminate between insects 3 or 10 days old may not be problematic for epidemiological considerations.

In Mayagaya et al. [36], the authors achieved 80% correct predictions for females when they were separated into two groups, young vs. old. They also mention the difficulty of separating younger mosquitoes with a few days of difference (e.g. 1 day vs. 3 or 7 days old), consistent with our results for 3-day-old sand flies (Table 1a). In addition, in this reference, the authors had an accuracy of only 50% for males, which is also in accordance with our results when we analyzed six different age groups (Table 1), as we had better hits and accuracy for females than males. A similar result was also observed in *Drosophila* spp. [4].

In general, we observed lower accuracy and hits in males compared to females (Table 1 and 2). This fact may be attributed to males' small body size, which may result in a lower capture of structural information by infrared spectroscopy. This may affect physiological information too; for example, males ingest a smaller amount of sugar solution compared to females (13.1 ± 1.5 vs. 20.4 ± 1.9 nl) [19]. Another reason may be due to lesser changes in the cuticle of males than in the females, which may suffer physiological modifications related to blood feeding and oogenesis [36].

An improvement in age determination results was also observed when mated insects were classified only in two groups (young vs. old) compared to unmated ones (Table 2). This suggests that mated sand flies can provide more information about their chemical composition or structural organization. In addition, using mated insects brings more robustness to the technique, since this would be closer to field conditions.

Literature data comparing the age of dipterans in different conditions (wild populations and infection status) [58, 59], preservation protocols [55, 57], and exposure to insecticides [56] showed that even under different settings it is possible to achieve accuracy values and correct predictions > 80%.

Estimating age in insects is a complicated process and depends on diverse features. Modifications that occurred in the cuticle, in surface lipids, muscle tissue, and fat body, and in the reproductive or digestive systems, and other biochemical changes, may influence the absorbance of the spectra, and all this will be detected by NIRS [44]. Overall, our results are promising compared to previous prediction techniques such as ovarian dissection, assessment of pteridine accumulation, cuticular hydrocarbons, or evaluation of transcriptional profiles, since these techniques require more time and cost, as reported by other authors [36]. Confirming this, Shang et al. [51] show that age and temperature influence the composition of cuticle HC, affecting the absorbance in infrared, effectively predicting the age of pupae of *Sarcophaga peregrina*. The authors also reported that the infrared technique is equivalent to the analysis of cuticular hydrocarbons carried out by gas chromatography and mass spectrometry.

Interestingly, NIR spectroscopy was very efficient in classifying mated or unmated sand flies since we have accuracies > 98% even in a lower number of samples (Table 3). The figure shows us a clear separation between samples in PCA and LDA. These data indicate that copulation might cause many changes in the physiology of different parts of the sand fly's body once the scanning probe has been positioned more precisely on the head and thorax. One possible explanation for the high differentiation between groups in females is because mated females are fertilized and in a pre-oviposition state, awaiting the opportunity to feed on blood and continue development and egg laying.

This hypothesis was shown in *Drosophila melanogaster*, whose composition and quantity of hydrocarbons are influenced by reproductive status. This happens when females, after copulation, receive cuticular and semen hydrocarbons, consequently altering their chemical profile compared to a virgin female [7]. HCs act as sexual, species, age, nutritional, reproductive, and microbiome status signals [51].

According to Detinova [15], a high proportion of parous females (those who have already produced offspring) in an area of adult control measures, for example, indicates that the treatment has not been adequate. On the other hand, in an area with larval control measures, if the proportion of nulliparous females (those who have not produced offspring) is high, this signals that the reproduction process remains constant, generating new individuals, and hence the unsuccessfulness of control measures applied to immature stages. In addition, this kind of data has great relevance in endemic areas, since it provides an estimate of the population density of mated females that will look for a vertebrate host for blood-feeding.

Surprisingly, our results on the difference in the type of meal (sugar vs. blood) and gonotrophic cycle had a very low ratio of correct predictions and accuracy, especially in females that fed only on sugar solution. Mayagaya et al. [36] compared unfed, fed, and gravid female mosquitoes, and the accuracy they found for gravid was only 65% of correct classifications, similar to our result. This differs from the results obtained using NIRS in *A. aegypti* by Hall et al. [24] to measure the volume of blood ingested by mosquitoes. These low accuracies suggest that the age of those females (9–10 days) may be affecting the spectra, possibly because of the dehydration already reported in older insects [44]. Moreover, it was possible to observe the loss of quality of spectra in older sand flies, mainly > 15 days during the collection of the sand flies for the longevity tests, according to what was described previously. Liebman et al. [35] also found a low ratio of correct predictions (66.7%) for female *A. aegypti* that only fed on a sugar solution. These authors considered that the nutritional factor was influencing the mosquitoes’ size and quality of analysis. Therefore, our data may have been affected by age and diet, since 10-day-old females had already completed digestion of blood and oviposition, so it was not expected to have HC from the insemination of males, for example, molecules that NIRS could detect.

Another possible explanation for the low prediction results may be the absence of sample variability. Some authors emphasize the importance of external validation with wild samples to ensure better calibration models; in other words, a model should be more widespread to consider the particularities of each sample [32].

In this work, using NIRS with sand flies, we demonstrated some possibilities of using the technique for this vector. We had promising results for the estimate of the age of sand flies in laboratory conditions, especially in the classification of young (< 8d) and old (> 8d) insects, data with clear epidemiological relevance. This information may be the basis for further longevity studies in field populations and better evaluation of both surveillance and vector control strategies. Extending this technique to field populations is necessary to develop calibration models that include sand flies with variable nutritional and background conditions. We also showed that the technique was very sensitive in separating mated from non-mated insects, and experimental adjustments are necessary to better classify females through the gonotrophic cycle and type of feeding.

## Conclusions

This is the first time to our knowledge that NIRS was used to characterize sand fly individuals. NIRS can be used to discriminate between young (< 8 days old) and old (> 8 days old) adult *L. longipalpis* sand flies. NIRS can also be used for determining the copulation status of both sexes (mated vs. non-mated). The technique may be used in the future for studies of age structure of field populations and assessment of the impact of vector control strategies, aiming to shorten the life span or lower reproductive fitness. These features are critical for the vectorial capacity of sand flies and need to be considered in designing strategies for blocking *Leishmania* parasite transmission.

### Supplementary Information

**Additional file 1: Figure S1.** Principal component analysis (PCA) of non-copulated *Lutzomyia longipalpis* females at different ages. (a) One (blue squares), 3 (brown triangles), 8 (blue stars), 10 (red circles), 15 (green triangles), and 17 (light blue diamonds) days old; (b) 1 (blue squares), 8 (red circles), and 15 (green triangles) days old; (c) < 8 (blue squares) or ≥ 8 (red circles) days old. **Figure S2.** Loadings of PCA [principal component 1 (PC – 1)] of non-copulated *Lutzomyia longipalpis* females at different ages: (a) 1-8-15 days old and (b) < 8 or ≥ 8 days old.** Figure S3.** Loadings of PCA [principal component 1 (PC – 1)] of non-copulated *Lutzomyia longipalpis* males at different ages: (a) 1-3-8-10-15-17 days old, (b) 1 8 15 days old, and (c) < 8 or ≥ 8 days old. **Figure S4.** Principal component analysis (PCA) of non-copulated *Lutzomyia longipalpis* males at different ages. (a) One (blue squares), 3 (brown triangles), 8 (gray stars), 10 (red circles), 15 (green triangles), and 17 (light blue diamonds) days old; (b) 1 (blue squares), 8 (red circles), and 15 (green triangles) days old; (c) < 8 (blue squares) or ≥ 8 (red circles) days old. **Figure S5.** Loadings of PCA [principal component 1 (PC – 1)] of copulated *Lutzomyia longipalpis* females at different ages: (a) 1-8-15 days old and (b) < 8 or > 8 days old, zoomed in. **Figure S6.** Loadings of PCA (principal component 1 (PC – 1)) of copulated *Lutzomyia longipalpis* males at different ages: (a) 1-8-15 days old and (b) < 8 or > 8 days old. **Figure S7.** Principal component analysis (PCA) of copulated *Lutzomyia longipalpis* at different ages. (a) One (blue squares), 8 (green triangles), and 15 (red circles) days old; (b) < 8 (blue triangles) or > 8 (red circles) days old. **Figure S8.** Principal component analysis (PCA) of copulated *Lutzomyia longipalpis* males at different ages. (a) One (blue squares), 8(green triangles), and 15 (red circles) days old; (b) < 8 (blue squares) or > 8 (red circles) days old. **Figure S9.** Principal component analysis (PCA) of copulated (CP, blue squares) vs. non copulated (NC, red circles) *Lutzomyia longipalpis* at the same age. (a) Females; (b) males. **Figure S10.** Principal component analysis (PCA) of females fed on sucrose solution (Suc, blue squares) or sucrose plus blood (Suc_B, red circles). (a) Results keeping females with unlaid eggs, (b) without females with unlaid eggs.

## Data Availability

All data generated or analyzed during this study are included in this published article.
