# Supplementary figures and images for: Age, sex, and mating status discrimination in the sand fly Lutzomyia longipalpis using near infra-red spectroscopy (NIRS)
Source: Parasit Vectors. 2024 Jan 12;17:19. doi: 10.1186/s13071-023-06097-1 (PMC10787389; doi:10.1186/s13071-023-06097-1)

Scores

A

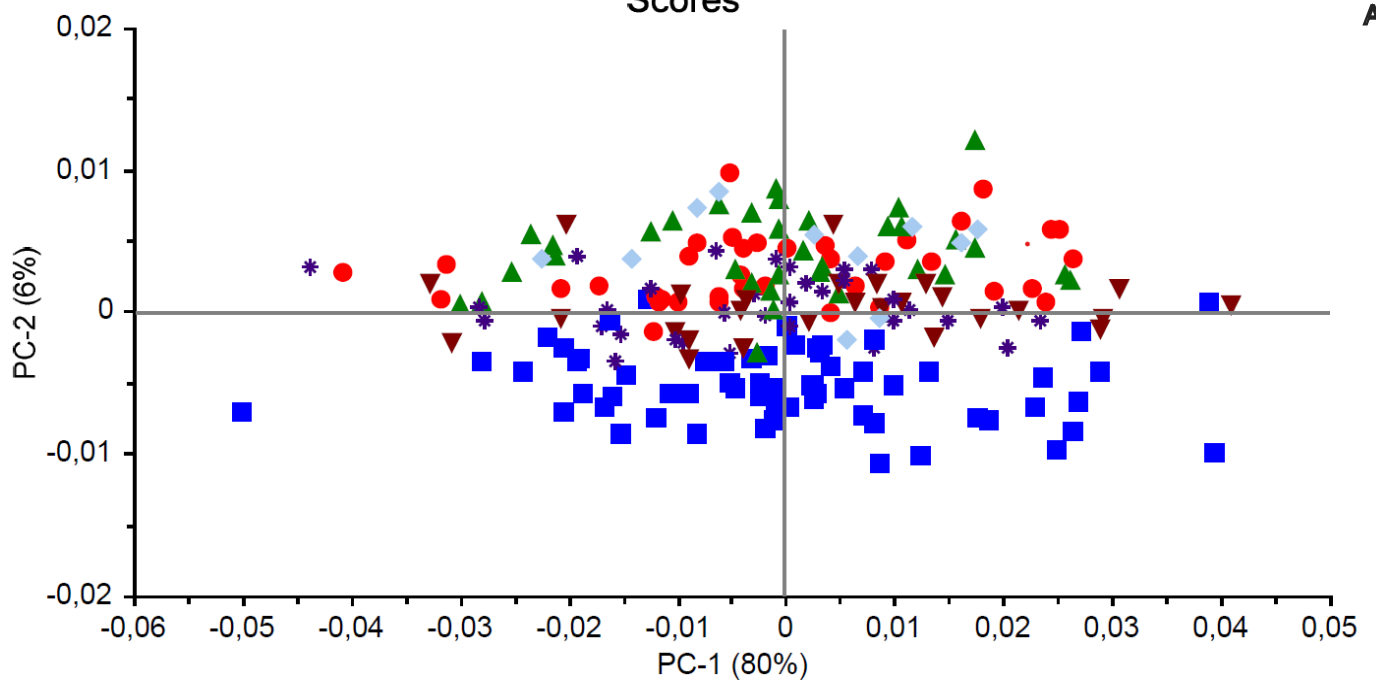

■ 1    ● 10    ▲ 15    ◆ 17    ▼ 3    \* 8

B

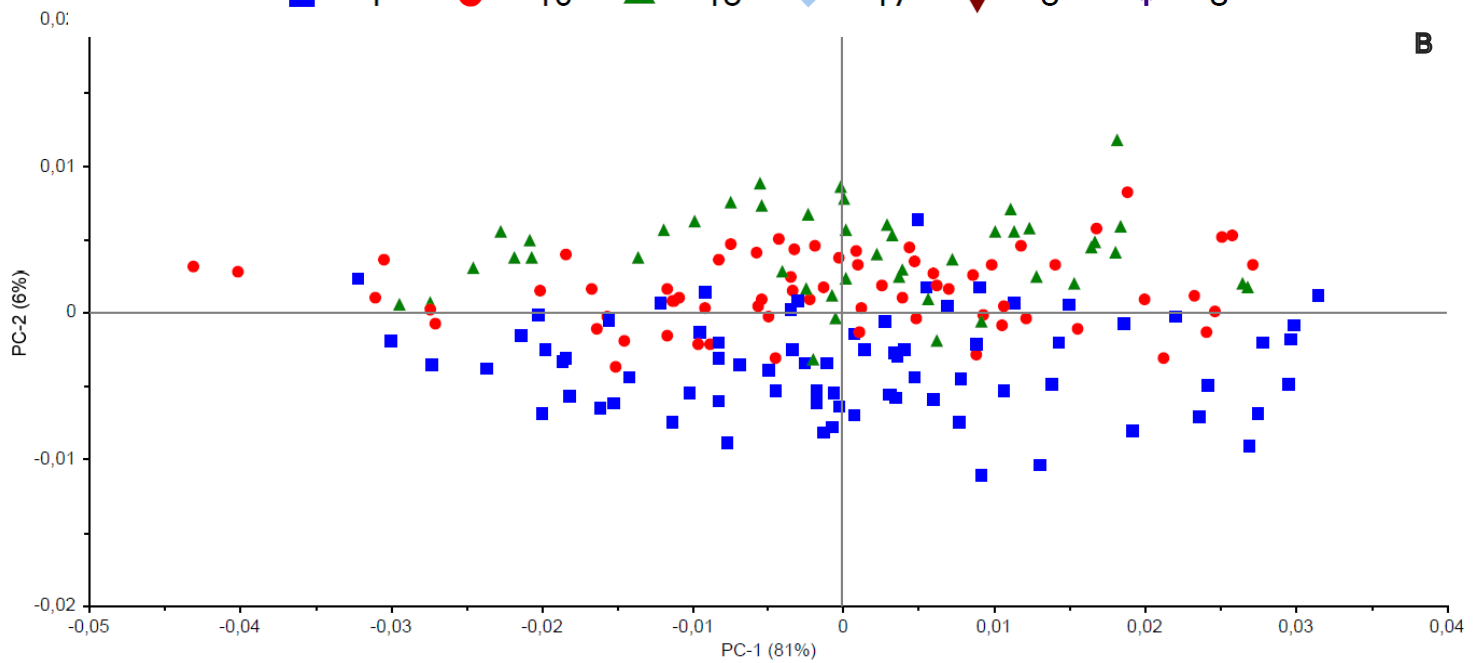

■ 1    ● 8    ▲ 15

C

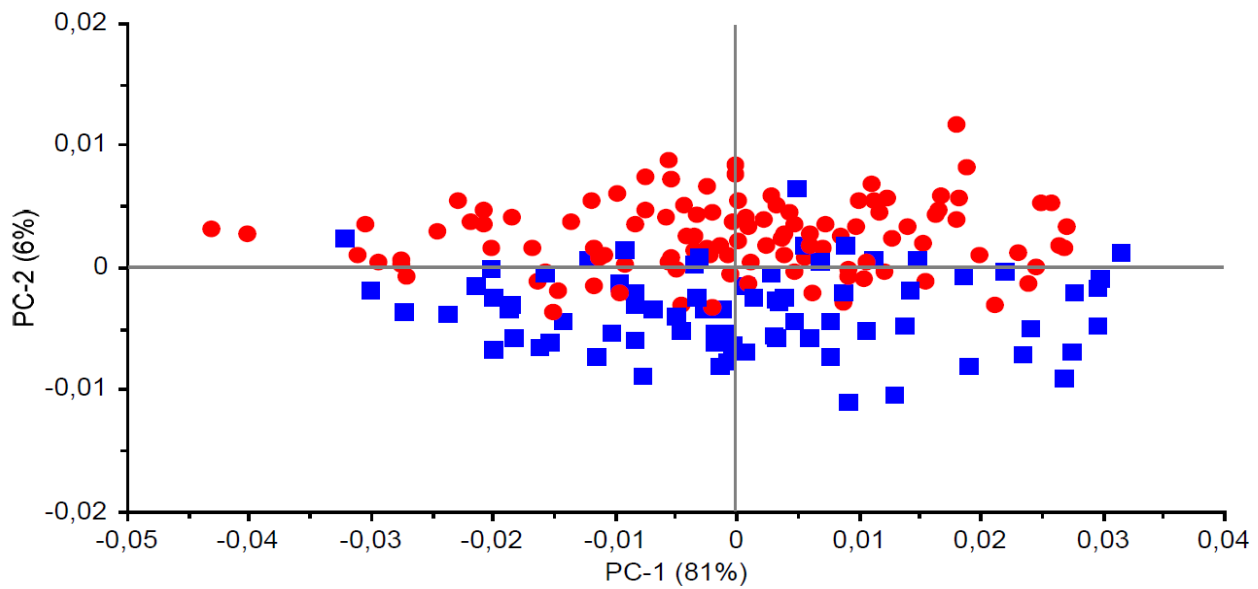

■ <8    ● ≥8

Supplement: Supplementary file 1 — Additional file 1: Figure S1. Principal component analysis (PCA) of non-copulated Lutzomyia longipalpis females at different ages. (a) One (blue squares), 3 (brown triangles), 8 (blue stars), 10 (red circles), 15 (green triangles), and 17 (light blue diamonds) days old; (b) 1 (blue squares), 8 (red circles), and 15 (green triangles) days old; (c) < 8 (blue squares) or ≥ 8 (red circles) days old. Figure S2. Loadings of PCA [principal component 1 (PC – 1)] of non-copulated Lutzomyia longipalpis females at different ages: (a) 1-8-15 days old and (b) < 8 or ≥ 8 days old. Figure S3. Loadings of PCA [principal component 1 (PC – 1)] of non-copulated Lutzomyia longipalpis males at different ages: (a) 1-3-8-10-15-17 days old, (b) 1 8 15 days old, and (c) < 8 or ≥ 8 days old. Figure S4. Principal component analysis (PCA) of non-copulated Lutzomyia longipalpis males at different ages. (a) One (blue squares), 3 (brown triangles), 8 (gray stars), 10 (red circles), 15 (green triangles), and 17 (light blue diamonds) days old; (b) 1 (blue squares), 8 (red circles), and 15 (green triangles) days old; (c) < 8 (blue squares) or ≥ 8 (red circles) days old. Figure S5. Loadings of PCA [principal component 1 (PC – 1)] of copulated Lutzomyia longipalpis females at different ages: (a) 1-8-15 days old and (b) < 8 or > 8 days old, zoomed in. Figure S6. Loadings of PCA (principal component 1 (PC – 1)) of copulated Lutzomyia longipalpis males at different ages: (a) 1-8-15 days old and (b) < 8 or > 8 days old. Figure S7. Principal component analysis (PCA) of copulated Lutzomyia longipalpis at different ages. (a) One (blue squares), 8 (green triangles), and 15 (red circles) days old; (b) < 8 (blue triangles) or > 8 (red circles) days old. Figure S8. Principal component analysis (PCA) of copulated Lutzomyia longipalpis males at different ages. (a) One (blue squares), 8(green triangles), and 15 (red circles) days old; (b) < 8 (blue squares) or > 8 (red circles) days old. Figure S9. Principal compon [file 13071_2023_6097_MOESM1_ESM.zip › Supple fig/Supplementary Figure 1.pdf]

A

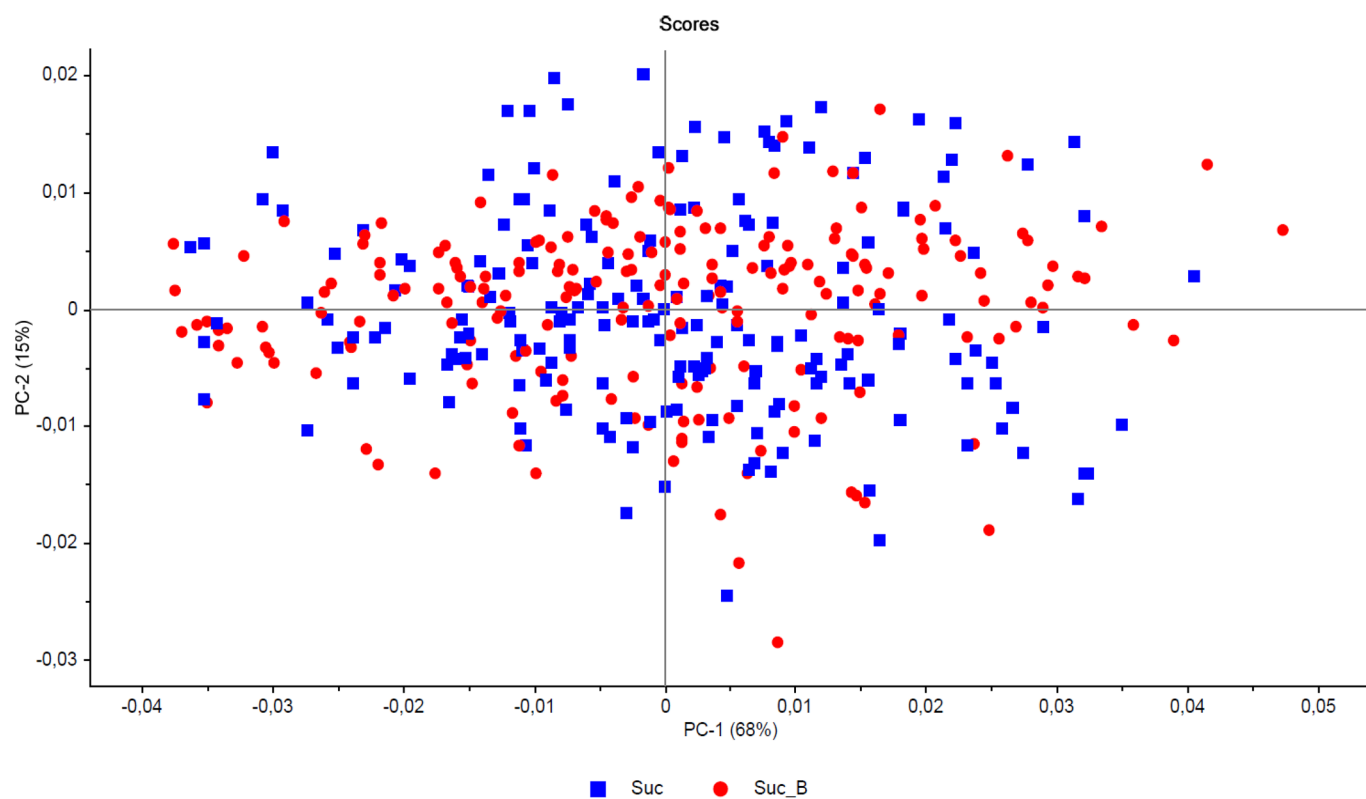

B

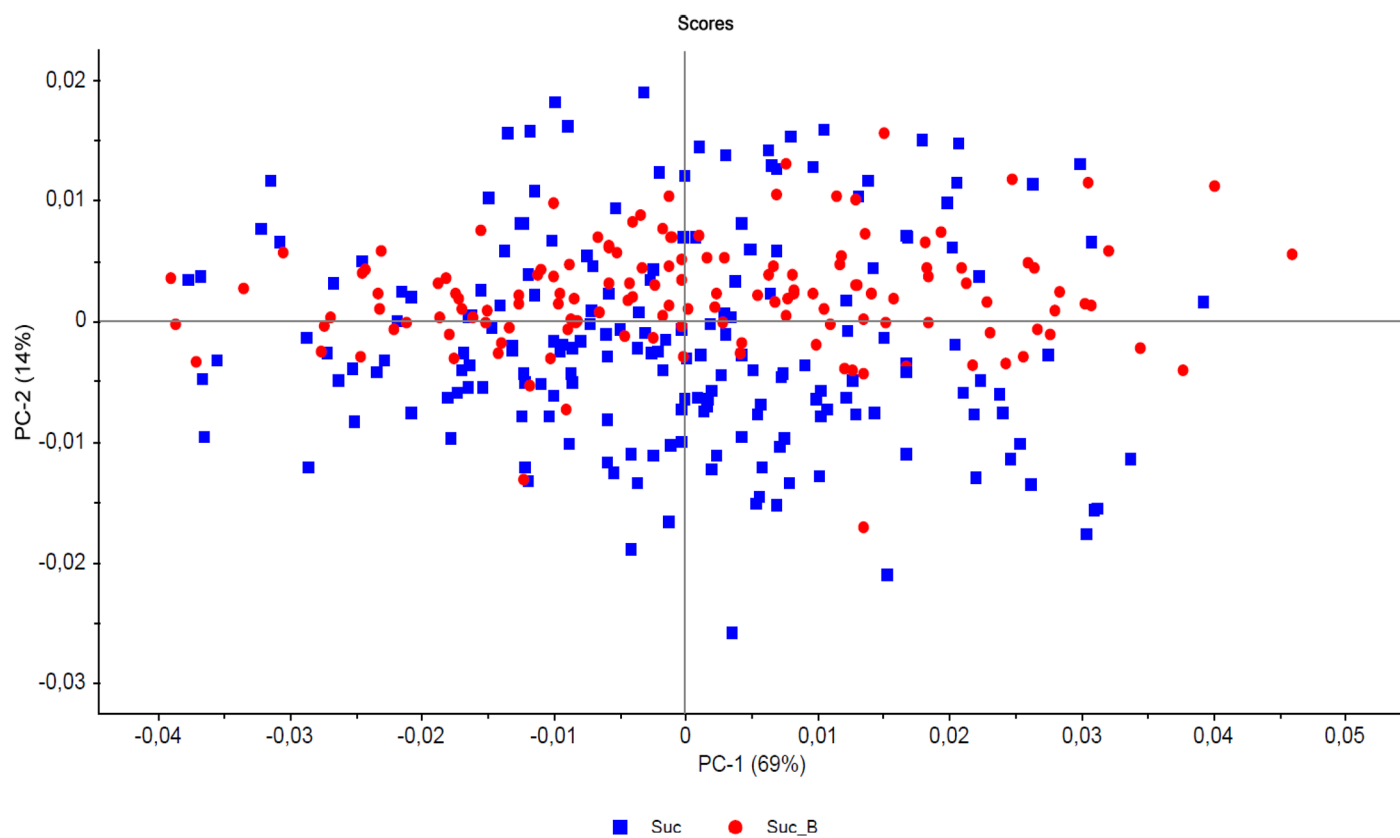

Supplement: Supplementary file 1 — Additional file 1: Figure S1. Principal component analysis (PCA) of non-copulated Lutzomyia longipalpis females at different ages. (a) One (blue squares), 3 (brown triangles), 8 (blue stars), 10 (red circles), 15 (green triangles), and 17 (light blue diamonds) days old; (b) 1 (blue squares), 8 (red circles), and 15 (green triangles) days old; (c) < 8 (blue squares) or ≥ 8 (red circles) days old. Figure S2. Loadings of PCA [principal component 1 (PC – 1)] of non-copulated Lutzomyia longipalpis females at different ages: (a) 1-8-15 days old and (b) < 8 or ≥ 8 days old. Figure S3. Loadings of PCA [principal component 1 (PC – 1)] of non-copulated Lutzomyia longipalpis males at different ages: (a) 1-3-8-10-15-17 days old, (b) 1 8 15 days old, and (c) < 8 or ≥ 8 days old. Figure S4. Principal component analysis (PCA) of non-copulated Lutzomyia longipalpis males at different ages. (a) One (blue squares), 3 (brown triangles), 8 (gray stars), 10 (red circles), 15 (green triangles), and 17 (light blue diamonds) days old; (b) 1 (blue squares), 8 (red circles), and 15 (green triangles) days old; (c) < 8 (blue squares) or ≥ 8 (red circles) days old. Figure S5. Loadings of PCA [principal component 1 (PC – 1)] of copulated Lutzomyia longipalpis females at different ages: (a) 1-8-15 days old and (b) < 8 or > 8 days old, zoomed in. Figure S6. Loadings of PCA (principal component 1 (PC – 1)) of copulated Lutzomyia longipalpis males at different ages: (a) 1-8-15 days old and (b) < 8 or > 8 days old. Figure S7. Principal component analysis (PCA) of copulated Lutzomyia longipalpis at different ages. (a) One (blue squares), 8 (green triangles), and 15 (red circles) days old; (b) < 8 (blue triangles) or > 8 (red circles) days old. Figure S8. Principal component analysis (PCA) of copulated Lutzomyia longipalpis males at different ages. (a) One (blue squares), 8(green triangles), and 15 (red circles) days old; (b) < 8 (blue squares) or > 8 (red circles) days old. Figure S9. Principal compon [file 13071_2023_6097_MOESM1_ESM.zip › Supple fig/Supplementary Figure 10.pdf]

Loadings

A

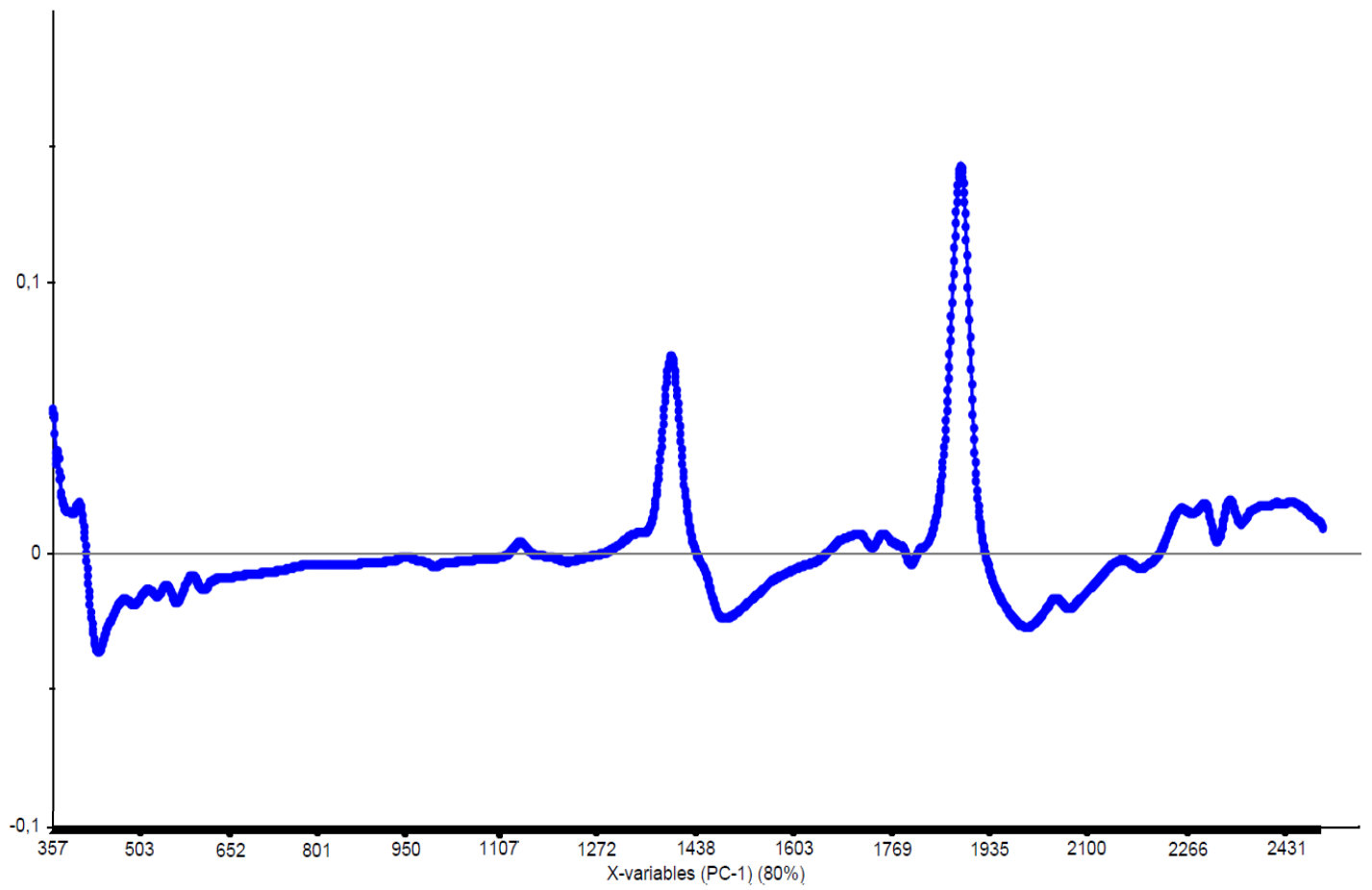

Loadings

B

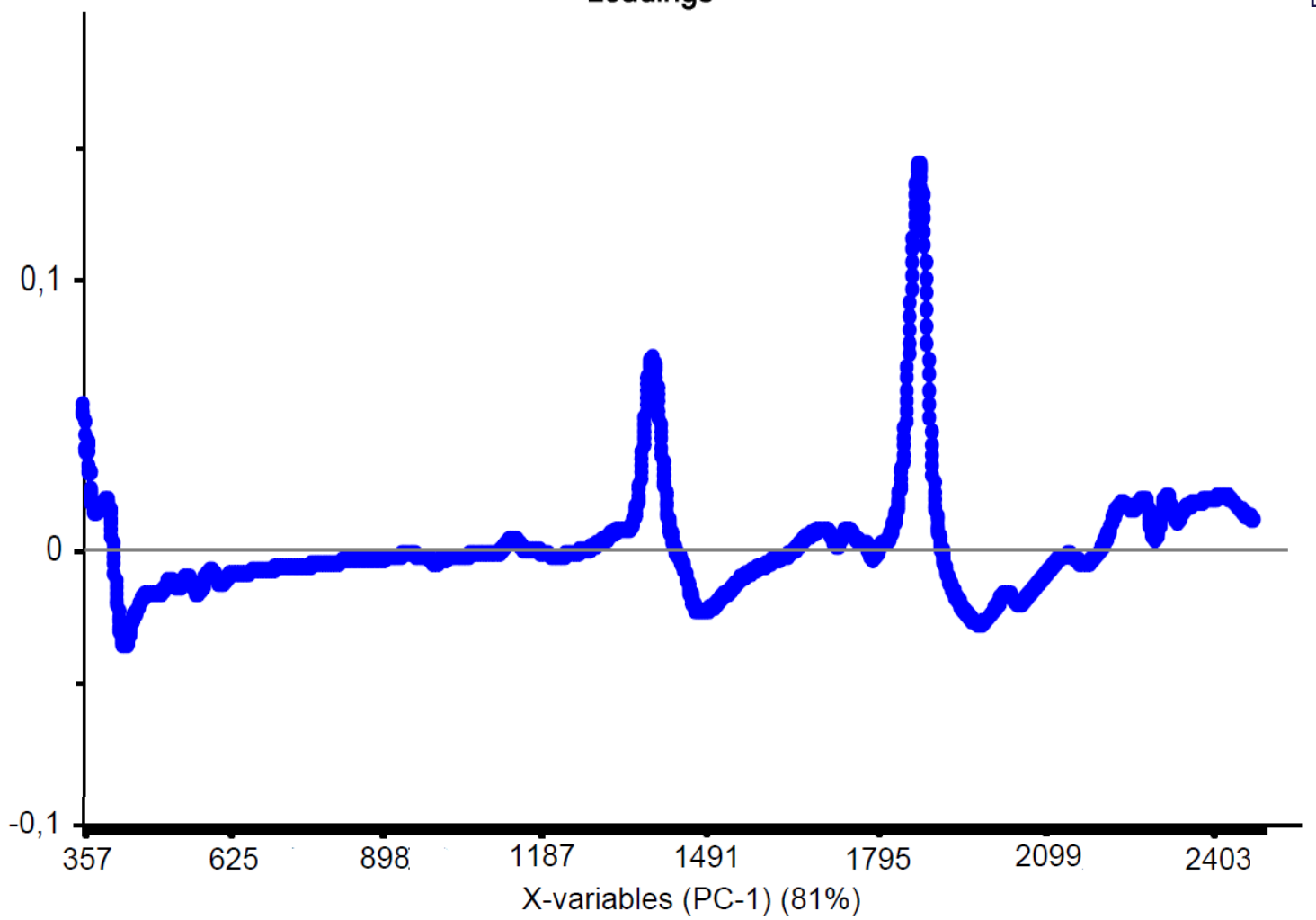

Supplement: Supplementary file 1 — Additional file 1: Figure S1. Principal component analysis (PCA) of non-copulated Lutzomyia longipalpis females at different ages. (a) One (blue squares), 3 (brown triangles), 8 (blue stars), 10 (red circles), 15 (green triangles), and 17 (light blue diamonds) days old; (b) 1 (blue squares), 8 (red circles), and 15 (green triangles) days old; (c) < 8 (blue squares) or ≥ 8 (red circles) days old. Figure S2. Loadings of PCA [principal component 1 (PC – 1)] of non-copulated Lutzomyia longipalpis females at different ages: (a) 1-8-15 days old and (b) < 8 or ≥ 8 days old. Figure S3. Loadings of PCA [principal component 1 (PC – 1)] of non-copulated Lutzomyia longipalpis males at different ages: (a) 1-3-8-10-15-17 days old, (b) 1 8 15 days old, and (c) < 8 or ≥ 8 days old. Figure S4. Principal component analysis (PCA) of non-copulated Lutzomyia longipalpis males at different ages. (a) One (blue squares), 3 (brown triangles), 8 (gray stars), 10 (red circles), 15 (green triangles), and 17 (light blue diamonds) days old; (b) 1 (blue squares), 8 (red circles), and 15 (green triangles) days old; (c) < 8 (blue squares) or ≥ 8 (red circles) days old. Figure S5. Loadings of PCA [principal component 1 (PC – 1)] of copulated Lutzomyia longipalpis females at different ages: (a) 1-8-15 days old and (b) < 8 or > 8 days old, zoomed in. Figure S6. Loadings of PCA (principal component 1 (PC – 1)) of copulated Lutzomyia longipalpis males at different ages: (a) 1-8-15 days old and (b) < 8 or > 8 days old. Figure S7. Principal component analysis (PCA) of copulated Lutzomyia longipalpis at different ages. (a) One (blue squares), 8 (green triangles), and 15 (red circles) days old; (b) < 8 (blue triangles) or > 8 (red circles) days old. Figure S8. Principal component analysis (PCA) of copulated Lutzomyia longipalpis males at different ages. (a) One (blue squares), 8(green triangles), and 15 (red circles) days old; (b) < 8 (blue squares) or > 8 (red circles) days old. Figure S9. Principal compon [file 13071_2023_6097_MOESM1_ESM.zip › Supple fig/Supplementary Figure 2.pdf]

A

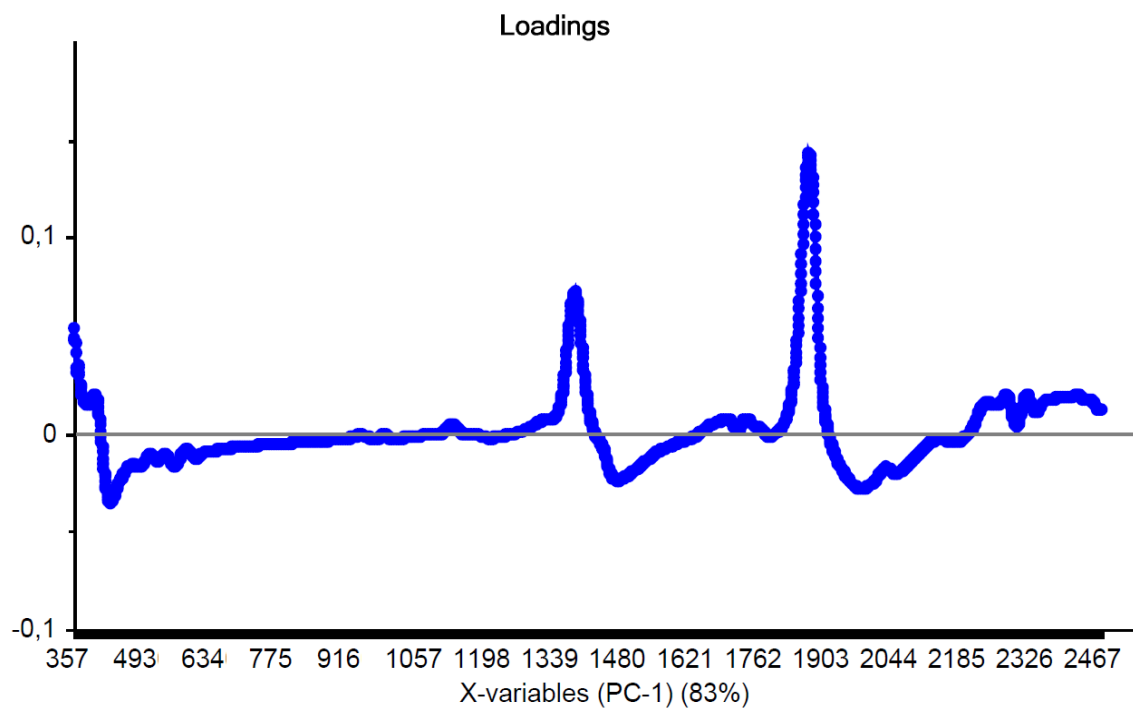

B

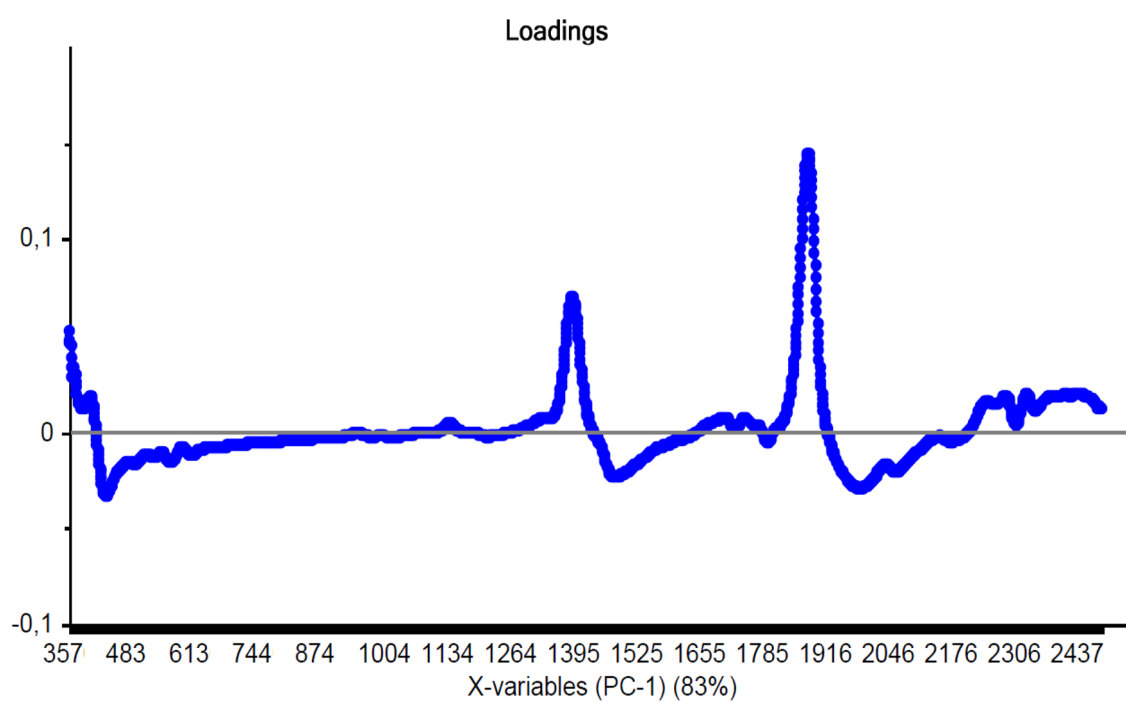

C

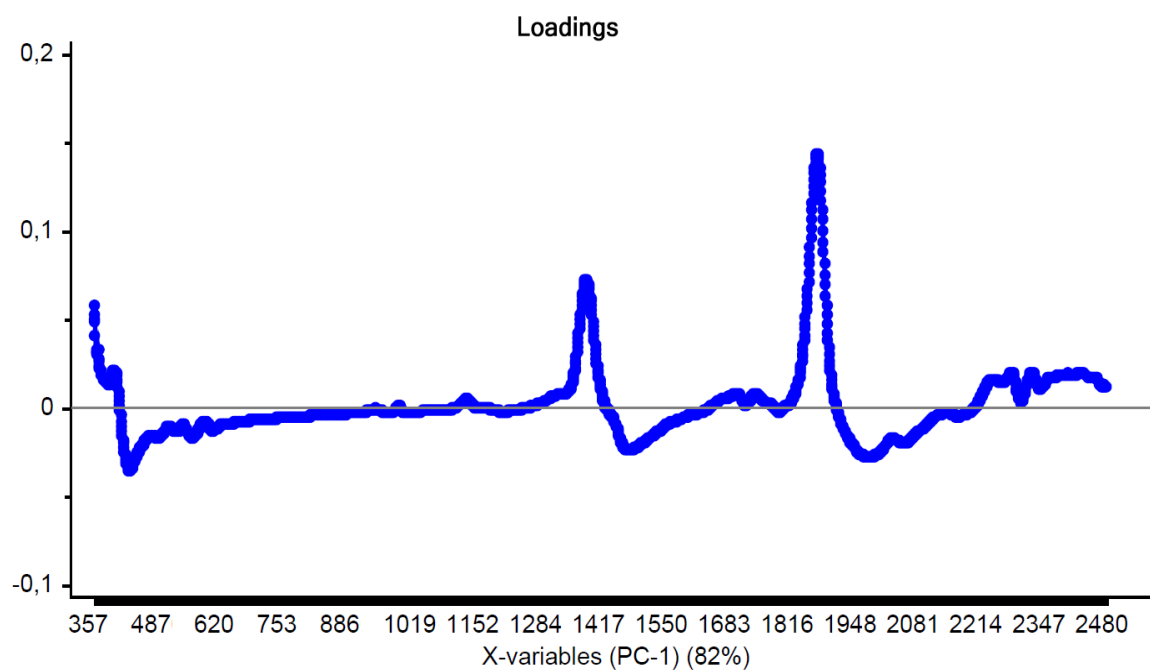

Supplement: Supplementary file 1 — Additional file 1: Figure S1. Principal component analysis (PCA) of non-copulated Lutzomyia longipalpis females at different ages. (a) One (blue squares), 3 (brown triangles), 8 (blue stars), 10 (red circles), 15 (green triangles), and 17 (light blue diamonds) days old; (b) 1 (blue squares), 8 (red circles), and 15 (green triangles) days old; (c) < 8 (blue squares) or ≥ 8 (red circles) days old. Figure S2. Loadings of PCA [principal component 1 (PC – 1)] of non-copulated Lutzomyia longipalpis females at different ages: (a) 1-8-15 days old and (b) < 8 or ≥ 8 days old. Figure S3. Loadings of PCA [principal component 1 (PC – 1)] of non-copulated Lutzomyia longipalpis males at different ages: (a) 1-3-8-10-15-17 days old, (b) 1 8 15 days old, and (c) < 8 or ≥ 8 days old. Figure S4. Principal component analysis (PCA) of non-copulated Lutzomyia longipalpis males at different ages. (a) One (blue squares), 3 (brown triangles), 8 (gray stars), 10 (red circles), 15 (green triangles), and 17 (light blue diamonds) days old; (b) 1 (blue squares), 8 (red circles), and 15 (green triangles) days old; (c) < 8 (blue squares) or ≥ 8 (red circles) days old. Figure S5. Loadings of PCA [principal component 1 (PC – 1)] of copulated Lutzomyia longipalpis females at different ages: (a) 1-8-15 days old and (b) < 8 or > 8 days old, zoomed in. Figure S6. Loadings of PCA (principal component 1 (PC – 1)) of copulated Lutzomyia longipalpis males at different ages: (a) 1-8-15 days old and (b) < 8 or > 8 days old. Figure S7. Principal component analysis (PCA) of copulated Lutzomyia longipalpis at different ages. (a) One (blue squares), 8 (green triangles), and 15 (red circles) days old; (b) < 8 (blue triangles) or > 8 (red circles) days old. Figure S8. Principal component analysis (PCA) of copulated Lutzomyia longipalpis males at different ages. (a) One (blue squares), 8(green triangles), and 15 (red circles) days old; (b) < 8 (blue squares) or > 8 (red circles) days old. Figure S9. Principal compon [file 13071_2023_6097_MOESM1_ESM.zip › Supple fig/Supplementary Figure 3.pdf]

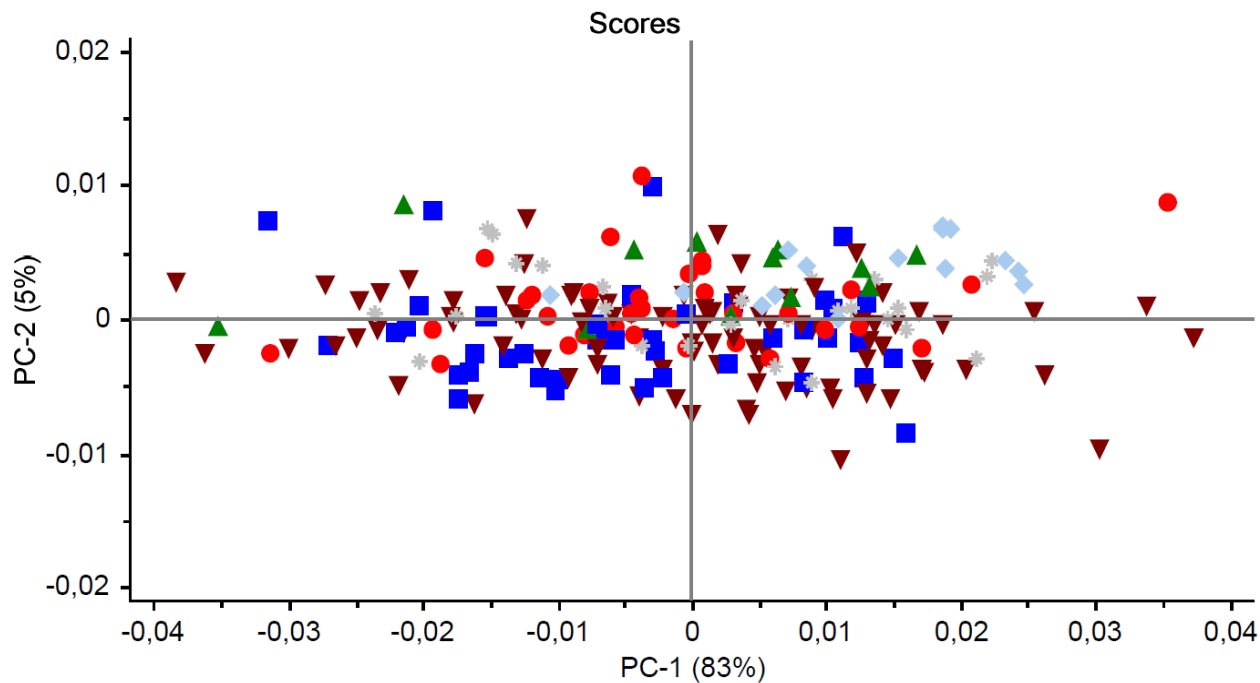

A

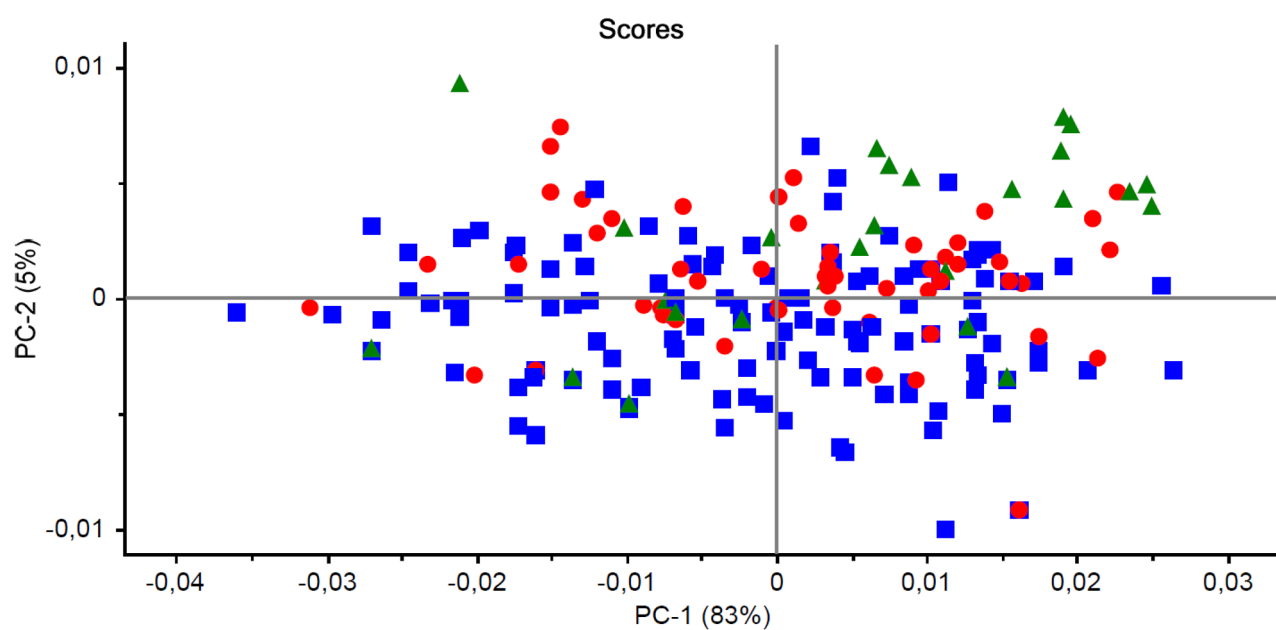

B

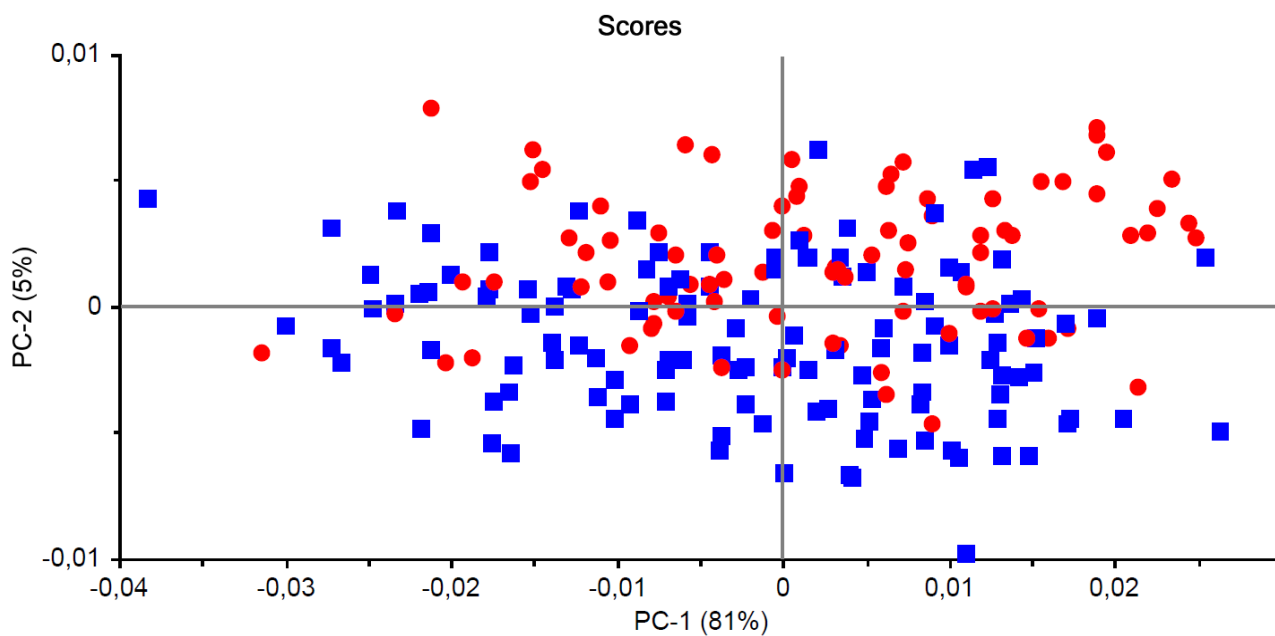

C

Supplement: Supplementary file 1 — Additional file 1: Figure S1. Principal component analysis (PCA) of non-copulated Lutzomyia longipalpis females at different ages. (a) One (blue squares), 3 (brown triangles), 8 (blue stars), 10 (red circles), 15 (green triangles), and 17 (light blue diamonds) days old; (b) 1 (blue squares), 8 (red circles), and 15 (green triangles) days old; (c) < 8 (blue squares) or ≥ 8 (red circles) days old. Figure S2. Loadings of PCA [principal component 1 (PC – 1)] of non-copulated Lutzomyia longipalpis females at different ages: (a) 1-8-15 days old and (b) < 8 or ≥ 8 days old. Figure S3. Loadings of PCA [principal component 1 (PC – 1)] of non-copulated Lutzomyia longipalpis males at different ages: (a) 1-3-8-10-15-17 days old, (b) 1 8 15 days old, and (c) < 8 or ≥ 8 days old. Figure S4. Principal component analysis (PCA) of non-copulated Lutzomyia longipalpis males at different ages. (a) One (blue squares), 3 (brown triangles), 8 (gray stars), 10 (red circles), 15 (green triangles), and 17 (light blue diamonds) days old; (b) 1 (blue squares), 8 (red circles), and 15 (green triangles) days old; (c) < 8 (blue squares) or ≥ 8 (red circles) days old. Figure S5. Loadings of PCA [principal component 1 (PC – 1)] of copulated Lutzomyia longipalpis females at different ages: (a) 1-8-15 days old and (b) < 8 or > 8 days old, zoomed in. Figure S6. Loadings of PCA (principal component 1 (PC – 1)) of copulated Lutzomyia longipalpis males at different ages: (a) 1-8-15 days old and (b) < 8 or > 8 days old. Figure S7. Principal component analysis (PCA) of copulated Lutzomyia longipalpis at different ages. (a) One (blue squares), 8 (green triangles), and 15 (red circles) days old; (b) < 8 (blue triangles) or > 8 (red circles) days old. Figure S8. Principal component analysis (PCA) of copulated Lutzomyia longipalpis males at different ages. (a) One (blue squares), 8(green triangles), and 15 (red circles) days old; (b) < 8 (blue squares) or > 8 (red circles) days old. Figure S9. Principal compon [file 13071_2023_6097_MOESM1_ESM.zip › Supple fig/Supplementary Figure 4.pdf]

**A**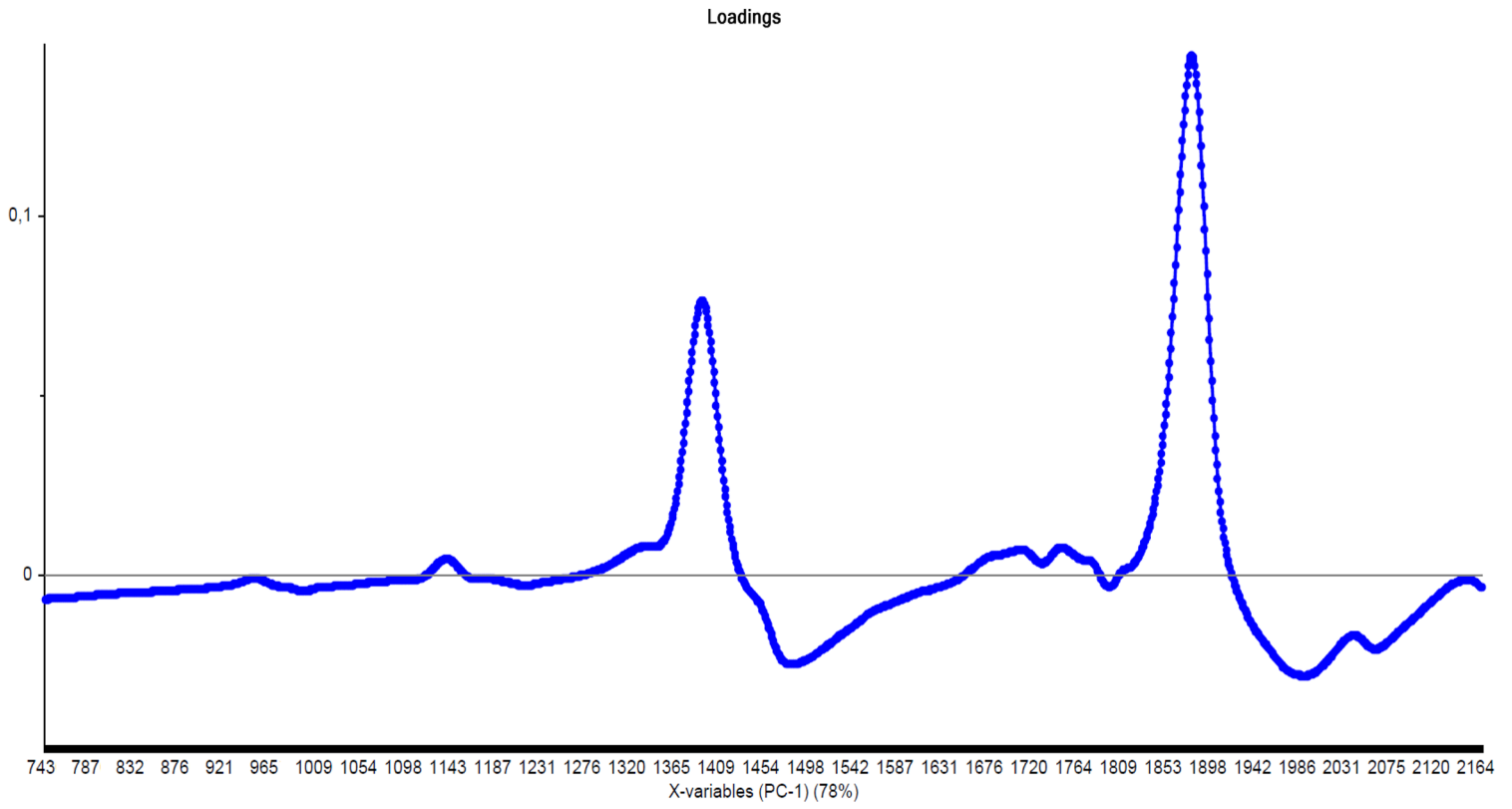**B**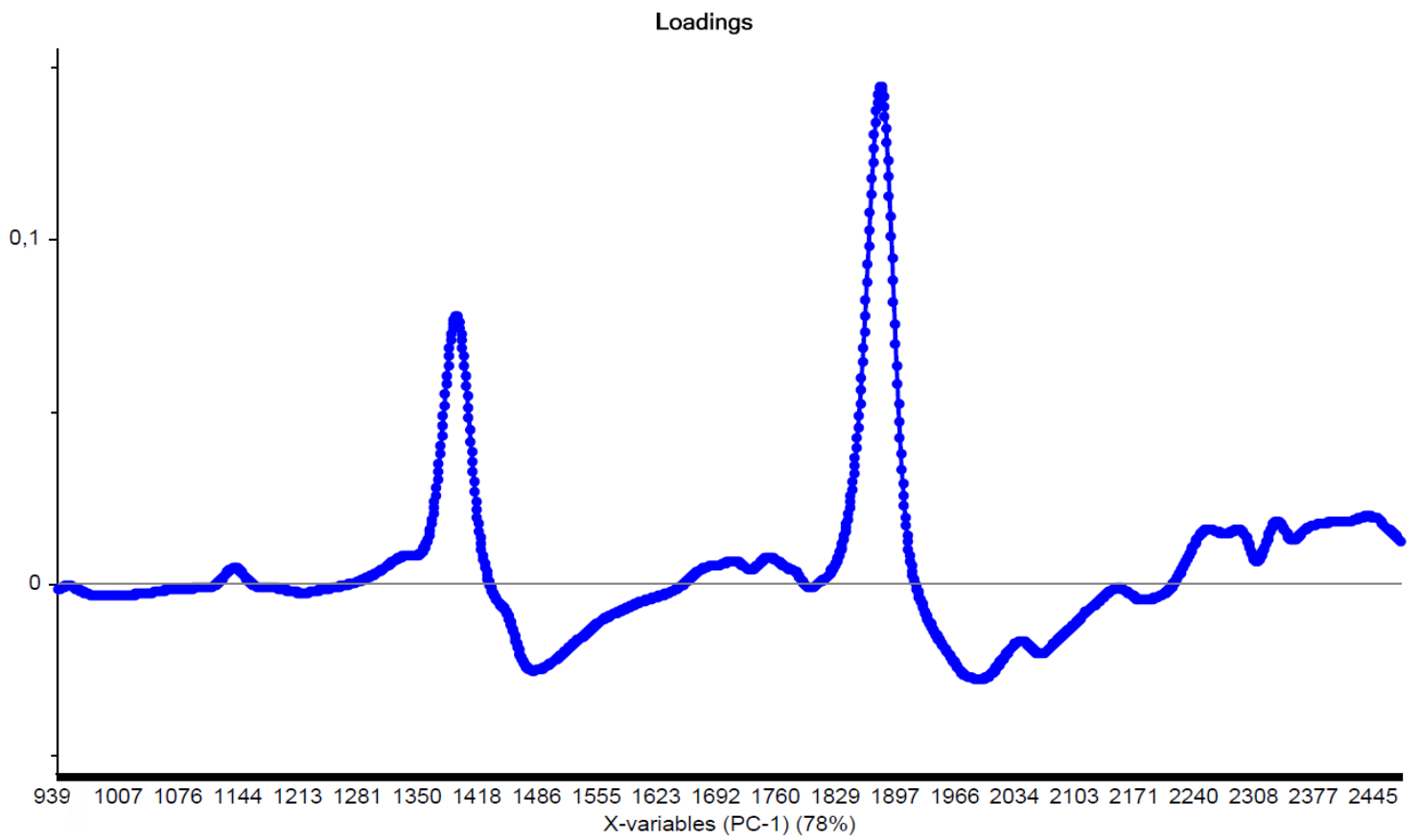

Supplement: Supplementary file 1 — Additional file 1: Figure S1. Principal component analysis (PCA) of non-copulated Lutzomyia longipalpis females at different ages. (a) One (blue squares), 3 (brown triangles), 8 (blue stars), 10 (red circles), 15 (green triangles), and 17 (light blue diamonds) days old; (b) 1 (blue squares), 8 (red circles), and 15 (green triangles) days old; (c) < 8 (blue squares) or ≥ 8 (red circles) days old. Figure S2. Loadings of PCA [principal component 1 (PC – 1)] of non-copulated Lutzomyia longipalpis females at different ages: (a) 1-8-15 days old and (b) < 8 or ≥ 8 days old. Figure S3. Loadings of PCA [principal component 1 (PC – 1)] of non-copulated Lutzomyia longipalpis males at different ages: (a) 1-3-8-10-15-17 days old, (b) 1 8 15 days old, and (c) < 8 or ≥ 8 days old. Figure S4. Principal component analysis (PCA) of non-copulated Lutzomyia longipalpis males at different ages. (a) One (blue squares), 3 (brown triangles), 8 (gray stars), 10 (red circles), 15 (green triangles), and 17 (light blue diamonds) days old; (b) 1 (blue squares), 8 (red circles), and 15 (green triangles) days old; (c) < 8 (blue squares) or ≥ 8 (red circles) days old. Figure S5. Loadings of PCA [principal component 1 (PC – 1)] of copulated Lutzomyia longipalpis females at different ages: (a) 1-8-15 days old and (b) < 8 or > 8 days old, zoomed in. Figure S6. Loadings of PCA (principal component 1 (PC – 1)) of copulated Lutzomyia longipalpis males at different ages: (a) 1-8-15 days old and (b) < 8 or > 8 days old. Figure S7. Principal component analysis (PCA) of copulated Lutzomyia longipalpis at different ages. (a) One (blue squares), 8 (green triangles), and 15 (red circles) days old; (b) < 8 (blue triangles) or > 8 (red circles) days old. Figure S8. Principal component analysis (PCA) of copulated Lutzomyia longipalpis males at different ages. (a) One (blue squares), 8(green triangles), and 15 (red circles) days old; (b) < 8 (blue squares) or > 8 (red circles) days old. Figure S9. Principal compon [file 13071_2023_6097_MOESM1_ESM.zip › Supple fig/Supplementary Figure 5.pdf]

**A**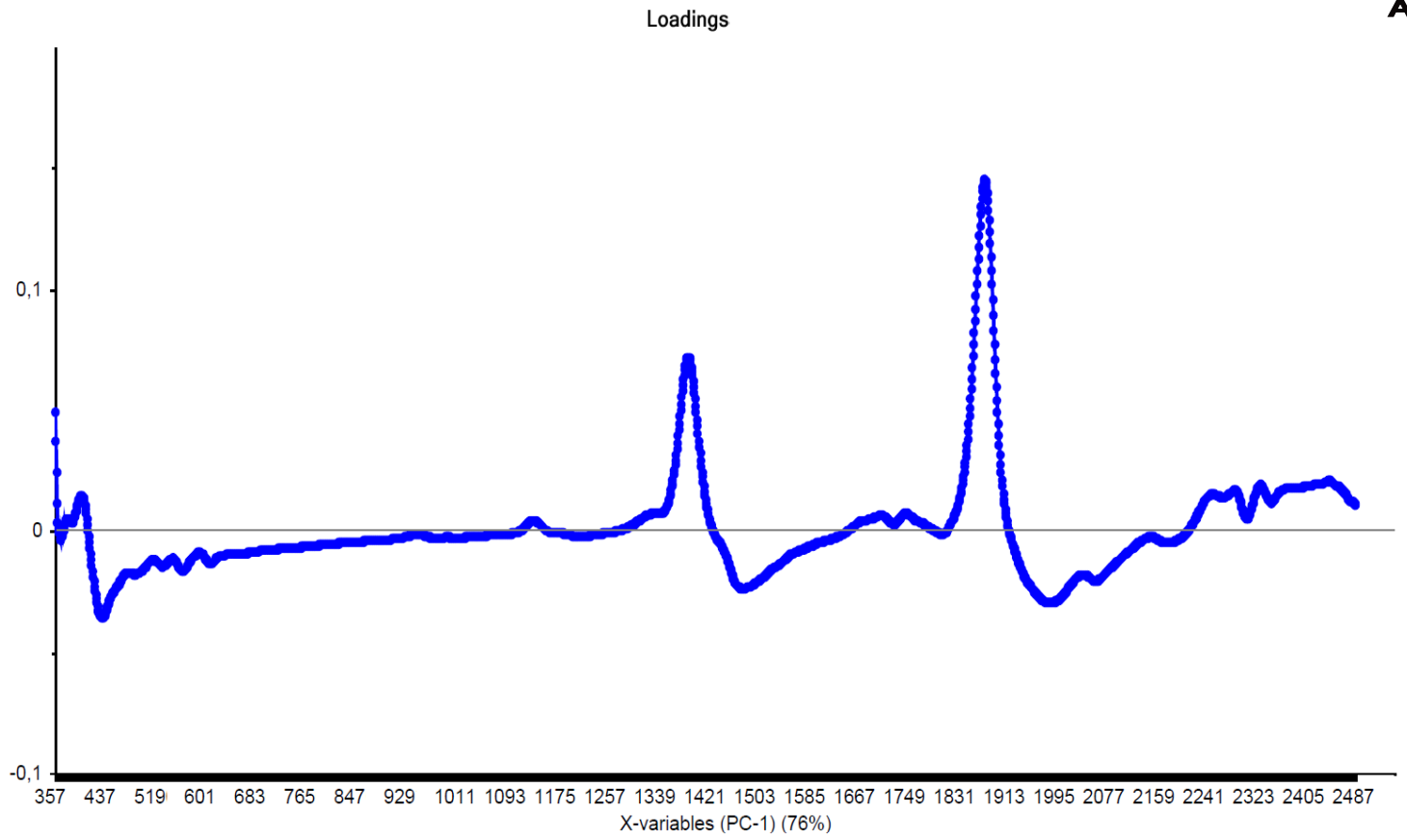**B**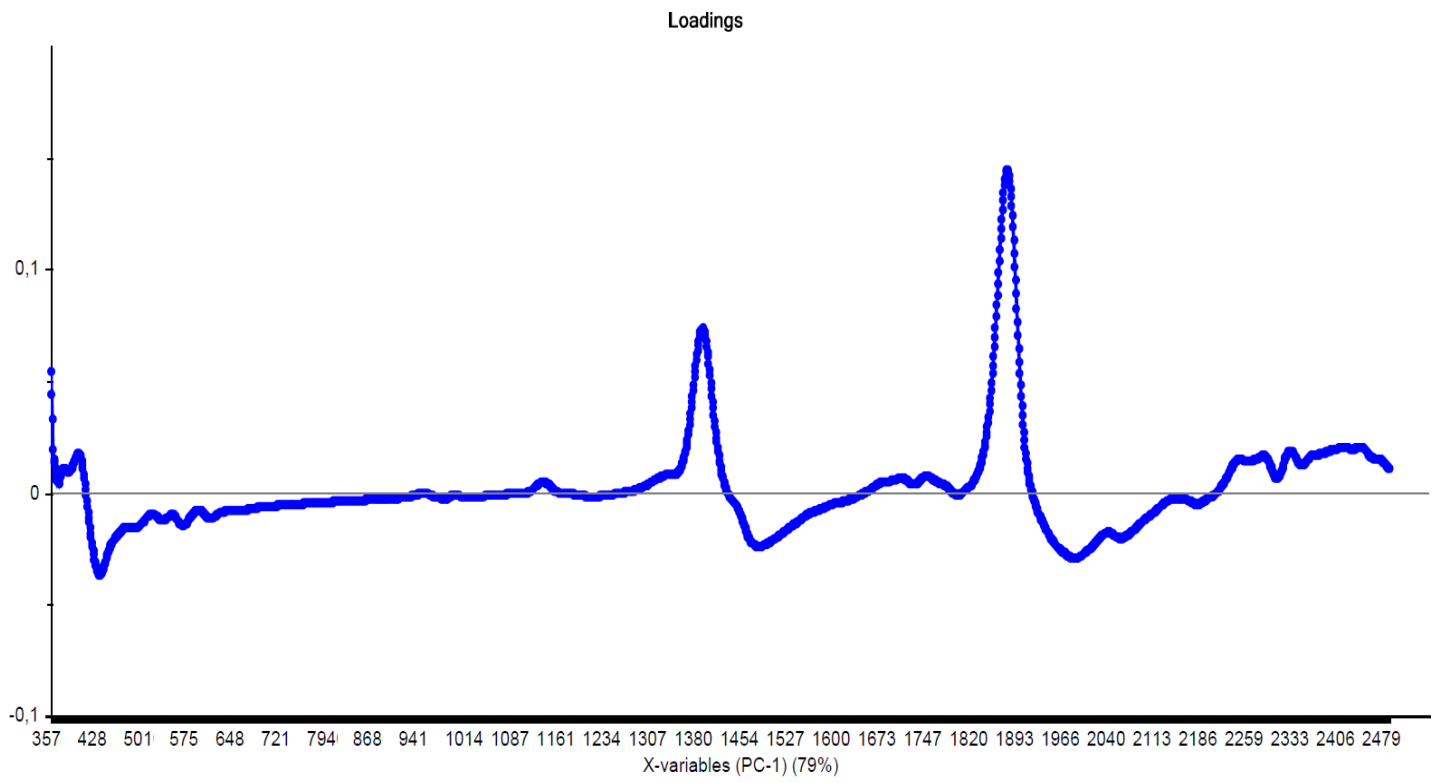

Supplement: Supplementary file 1 — Additional file 1: Figure S1. Principal component analysis (PCA) of non-copulated Lutzomyia longipalpis females at different ages. (a) One (blue squares), 3 (brown triangles), 8 (blue stars), 10 (red circles), 15 (green triangles), and 17 (light blue diamonds) days old; (b) 1 (blue squares), 8 (red circles), and 15 (green triangles) days old; (c) < 8 (blue squares) or ≥ 8 (red circles) days old. Figure S2. Loadings of PCA [principal component 1 (PC – 1)] of non-copulated Lutzomyia longipalpis females at different ages: (a) 1-8-15 days old and (b) < 8 or ≥ 8 days old. Figure S3. Loadings of PCA [principal component 1 (PC – 1)] of non-copulated Lutzomyia longipalpis males at different ages: (a) 1-3-8-10-15-17 days old, (b) 1 8 15 days old, and (c) < 8 or ≥ 8 days old. Figure S4. Principal component analysis (PCA) of non-copulated Lutzomyia longipalpis males at different ages. (a) One (blue squares), 3 (brown triangles), 8 (gray stars), 10 (red circles), 15 (green triangles), and 17 (light blue diamonds) days old; (b) 1 (blue squares), 8 (red circles), and 15 (green triangles) days old; (c) < 8 (blue squares) or ≥ 8 (red circles) days old. Figure S5. Loadings of PCA [principal component 1 (PC – 1)] of copulated Lutzomyia longipalpis females at different ages: (a) 1-8-15 days old and (b) < 8 or > 8 days old, zoomed in. Figure S6. Loadings of PCA (principal component 1 (PC – 1)) of copulated Lutzomyia longipalpis males at different ages: (a) 1-8-15 days old and (b) < 8 or > 8 days old. Figure S7. Principal component analysis (PCA) of copulated Lutzomyia longipalpis at different ages. (a) One (blue squares), 8 (green triangles), and 15 (red circles) days old; (b) < 8 (blue triangles) or > 8 (red circles) days old. Figure S8. Principal component analysis (PCA) of copulated Lutzomyia longipalpis males at different ages. (a) One (blue squares), 8(green triangles), and 15 (red circles) days old; (b) < 8 (blue squares) or > 8 (red circles) days old. Figure S9. Principal compon [file 13071_2023_6097_MOESM1_ESM.zip › Supple fig/Supplementary Figure 6.pdf]

**A**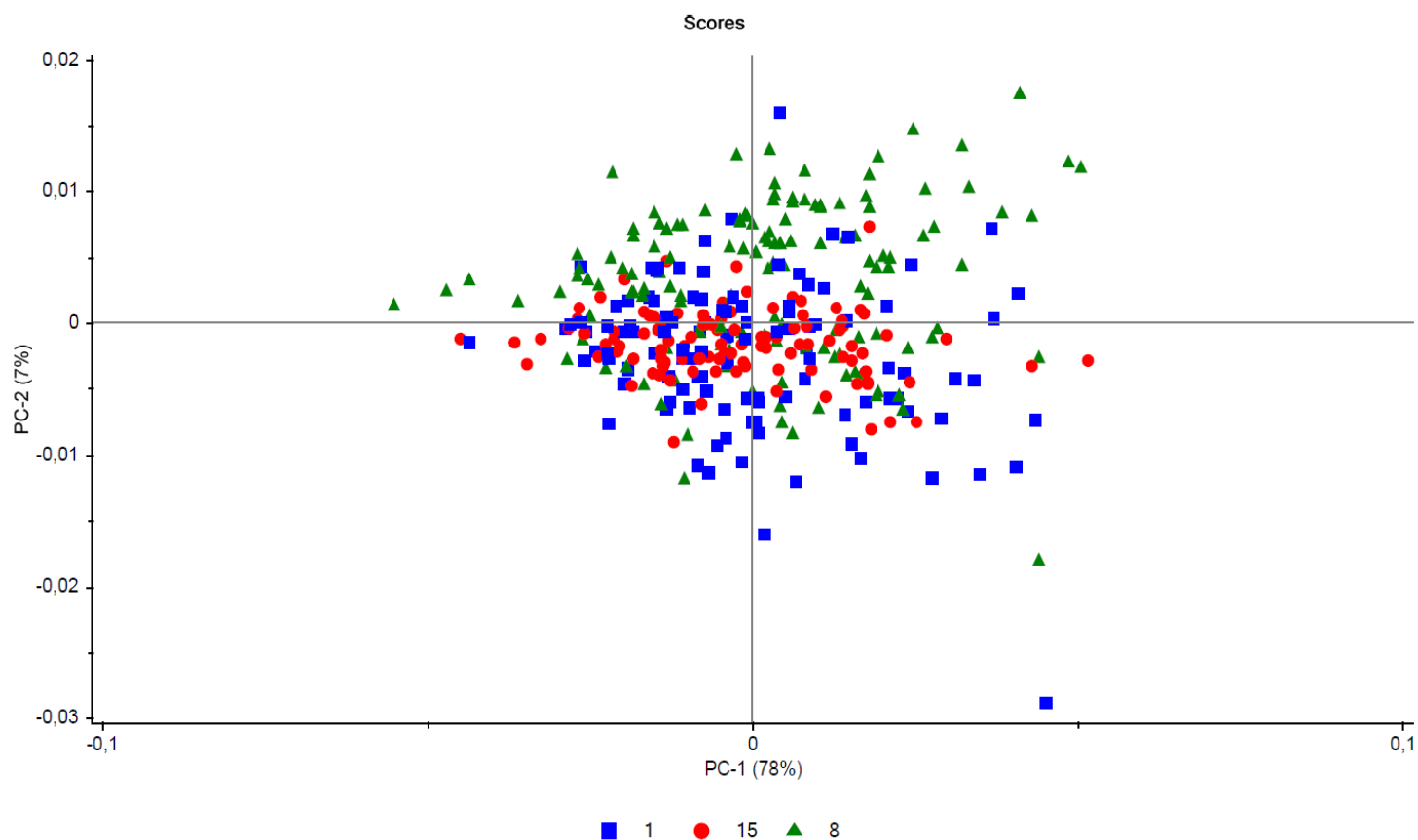**B**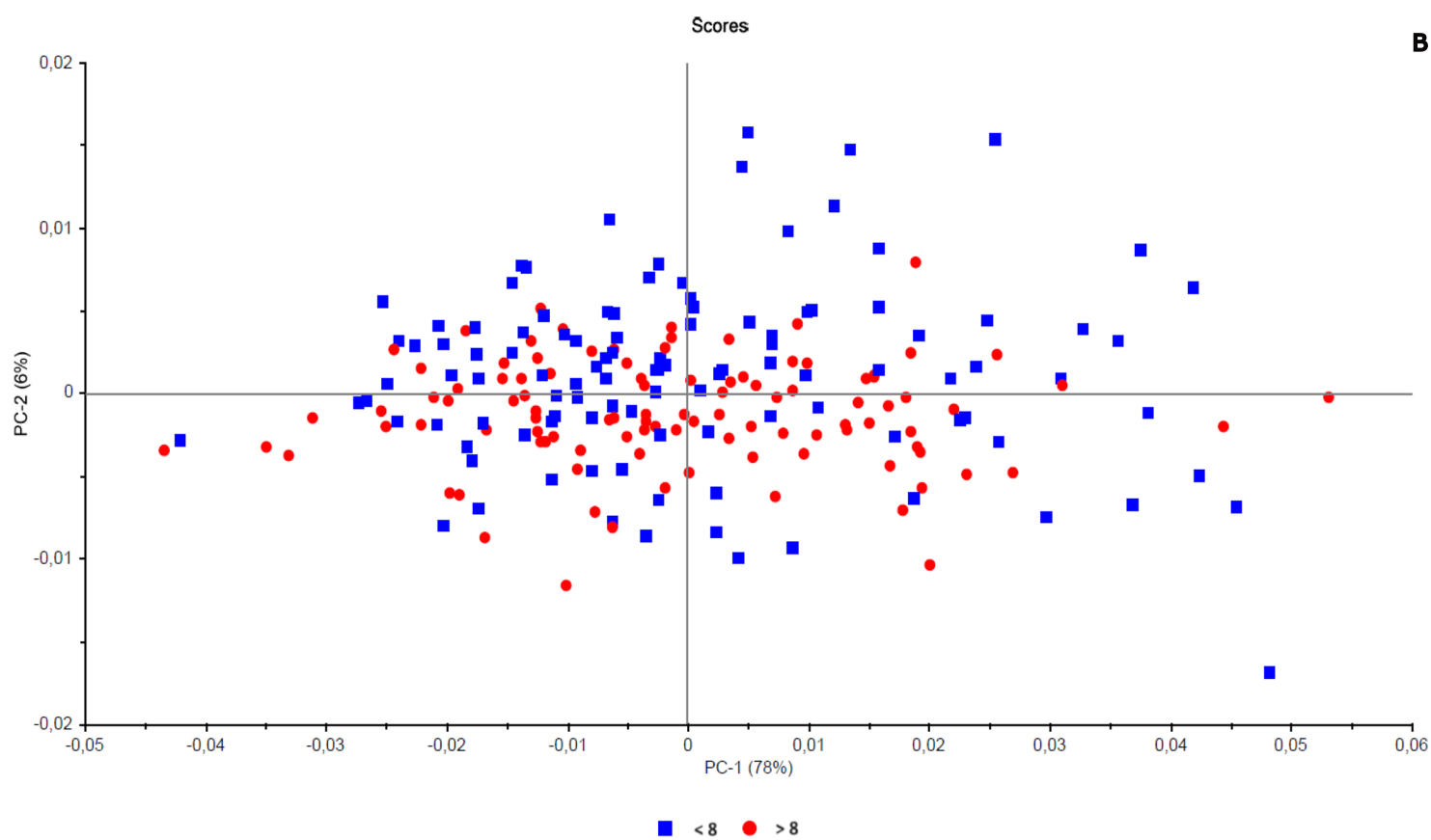

Supplement: Supplementary file 1 — Additional file 1: Figure S1. Principal component analysis (PCA) of non-copulated Lutzomyia longipalpis females at different ages. (a) One (blue squares), 3 (brown triangles), 8 (blue stars), 10 (red circles), 15 (green triangles), and 17 (light blue diamonds) days old; (b) 1 (blue squares), 8 (red circles), and 15 (green triangles) days old; (c) < 8 (blue squares) or ≥ 8 (red circles) days old. Figure S2. Loadings of PCA [principal component 1 (PC – 1)] of non-copulated Lutzomyia longipalpis females at different ages: (a) 1-8-15 days old and (b) < 8 or ≥ 8 days old. Figure S3. Loadings of PCA [principal component 1 (PC – 1)] of non-copulated Lutzomyia longipalpis males at different ages: (a) 1-3-8-10-15-17 days old, (b) 1 8 15 days old, and (c) < 8 or ≥ 8 days old. Figure S4. Principal component analysis (PCA) of non-copulated Lutzomyia longipalpis males at different ages. (a) One (blue squares), 3 (brown triangles), 8 (gray stars), 10 (red circles), 15 (green triangles), and 17 (light blue diamonds) days old; (b) 1 (blue squares), 8 (red circles), and 15 (green triangles) days old; (c) < 8 (blue squares) or ≥ 8 (red circles) days old. Figure S5. Loadings of PCA [principal component 1 (PC – 1)] of copulated Lutzomyia longipalpis females at different ages: (a) 1-8-15 days old and (b) < 8 or > 8 days old, zoomed in. Figure S6. Loadings of PCA (principal component 1 (PC – 1)) of copulated Lutzomyia longipalpis males at different ages: (a) 1-8-15 days old and (b) < 8 or > 8 days old. Figure S7. Principal component analysis (PCA) of copulated Lutzomyia longipalpis at different ages. (a) One (blue squares), 8 (green triangles), and 15 (red circles) days old; (b) < 8 (blue triangles) or > 8 (red circles) days old. Figure S8. Principal component analysis (PCA) of copulated Lutzomyia longipalpis males at different ages. (a) One (blue squares), 8(green triangles), and 15 (red circles) days old; (b) < 8 (blue squares) or > 8 (red circles) days old. Figure S9. Principal compon [file 13071_2023_6097_MOESM1_ESM.zip › Supple fig/Supplementary Figure 7.pdf]

A

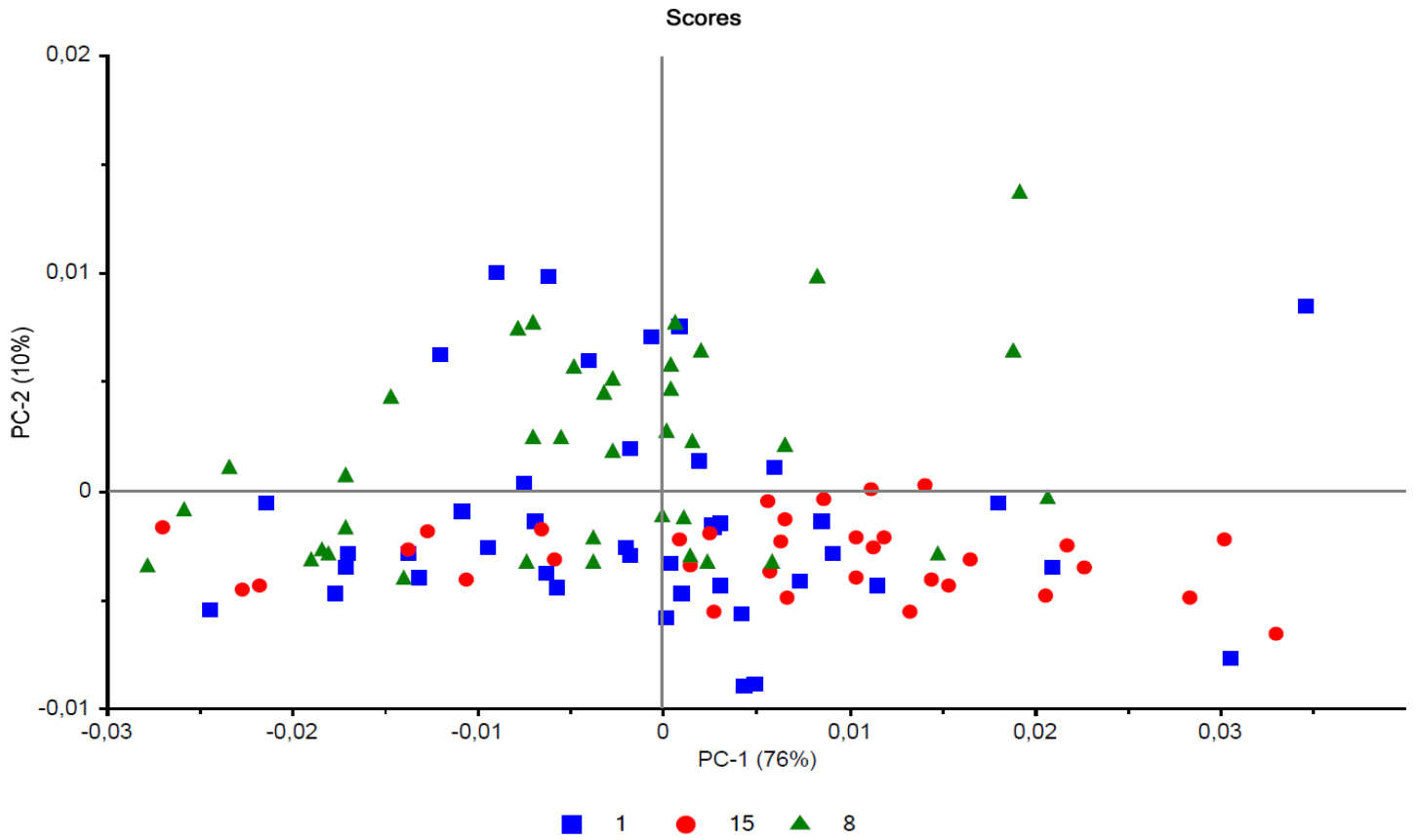

B

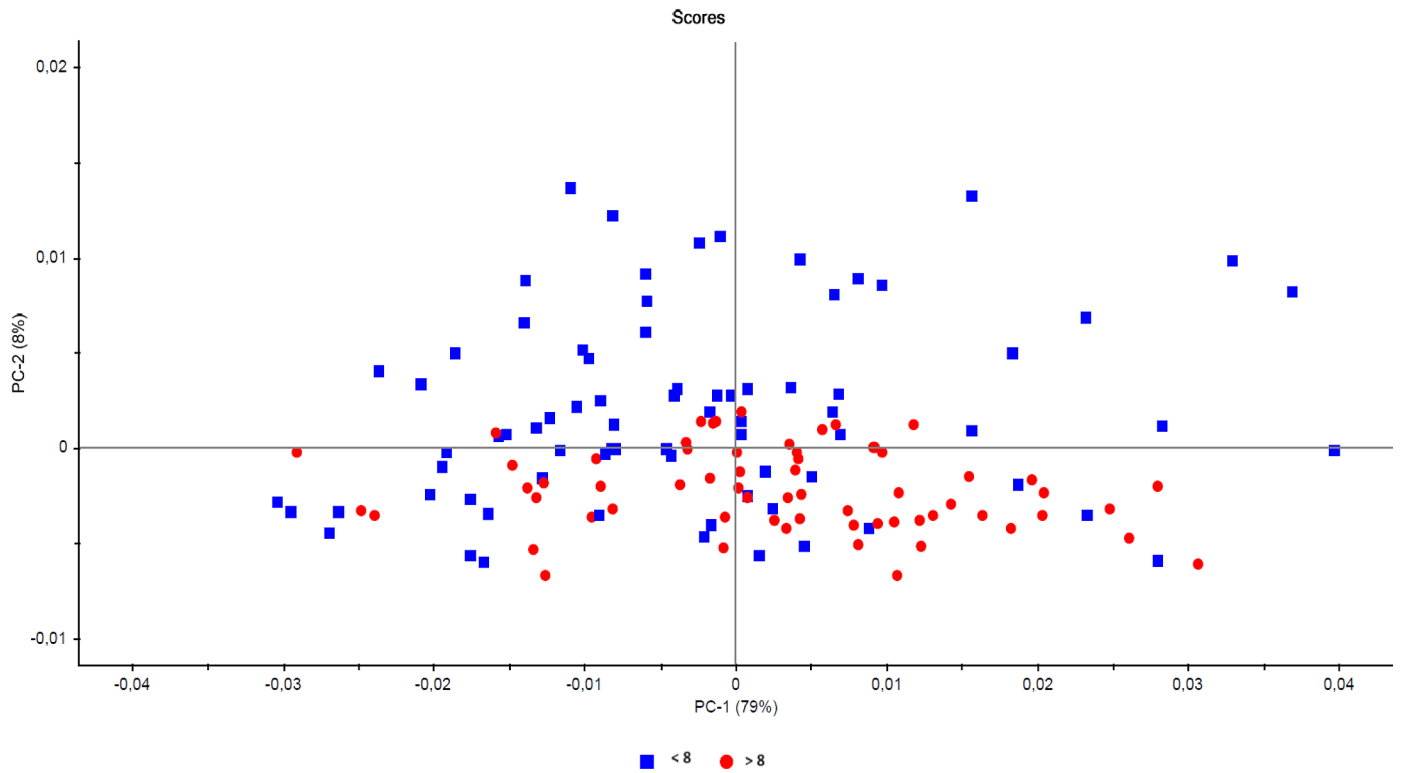

Supplement: Supplementary file 1 — Additional file 1: Figure S1. Principal component analysis (PCA) of non-copulated Lutzomyia longipalpis females at different ages. (a) One (blue squares), 3 (brown triangles), 8 (blue stars), 10 (red circles), 15 (green triangles), and 17 (light blue diamonds) days old; (b) 1 (blue squares), 8 (red circles), and 15 (green triangles) days old; (c) < 8 (blue squares) or ≥ 8 (red circles) days old. Figure S2. Loadings of PCA [principal component 1 (PC – 1)] of non-copulated Lutzomyia longipalpis females at different ages: (a) 1-8-15 days old and (b) < 8 or ≥ 8 days old. Figure S3. Loadings of PCA [principal component 1 (PC – 1)] of non-copulated Lutzomyia longipalpis males at different ages: (a) 1-3-8-10-15-17 days old, (b) 1 8 15 days old, and (c) < 8 or ≥ 8 days old. Figure S4. Principal component analysis (PCA) of non-copulated Lutzomyia longipalpis males at different ages. (a) One (blue squares), 3 (brown triangles), 8 (gray stars), 10 (red circles), 15 (green triangles), and 17 (light blue diamonds) days old; (b) 1 (blue squares), 8 (red circles), and 15 (green triangles) days old; (c) < 8 (blue squares) or ≥ 8 (red circles) days old. Figure S5. Loadings of PCA [principal component 1 (PC – 1)] of copulated Lutzomyia longipalpis females at different ages: (a) 1-8-15 days old and (b) < 8 or > 8 days old, zoomed in. Figure S6. Loadings of PCA (principal component 1 (PC – 1)) of copulated Lutzomyia longipalpis males at different ages: (a) 1-8-15 days old and (b) < 8 or > 8 days old. Figure S7. Principal component analysis (PCA) of copulated Lutzomyia longipalpis at different ages. (a) One (blue squares), 8 (green triangles), and 15 (red circles) days old; (b) < 8 (blue triangles) or > 8 (red circles) days old. Figure S8. Principal component analysis (PCA) of copulated Lutzomyia longipalpis males at different ages. (a) One (blue squares), 8(green triangles), and 15 (red circles) days old; (b) < 8 (blue squares) or > 8 (red circles) days old. Figure S9. Principal compon [file 13071_2023_6097_MOESM1_ESM.zip › Supple fig/Supplementary Figure 8.pdf]

A

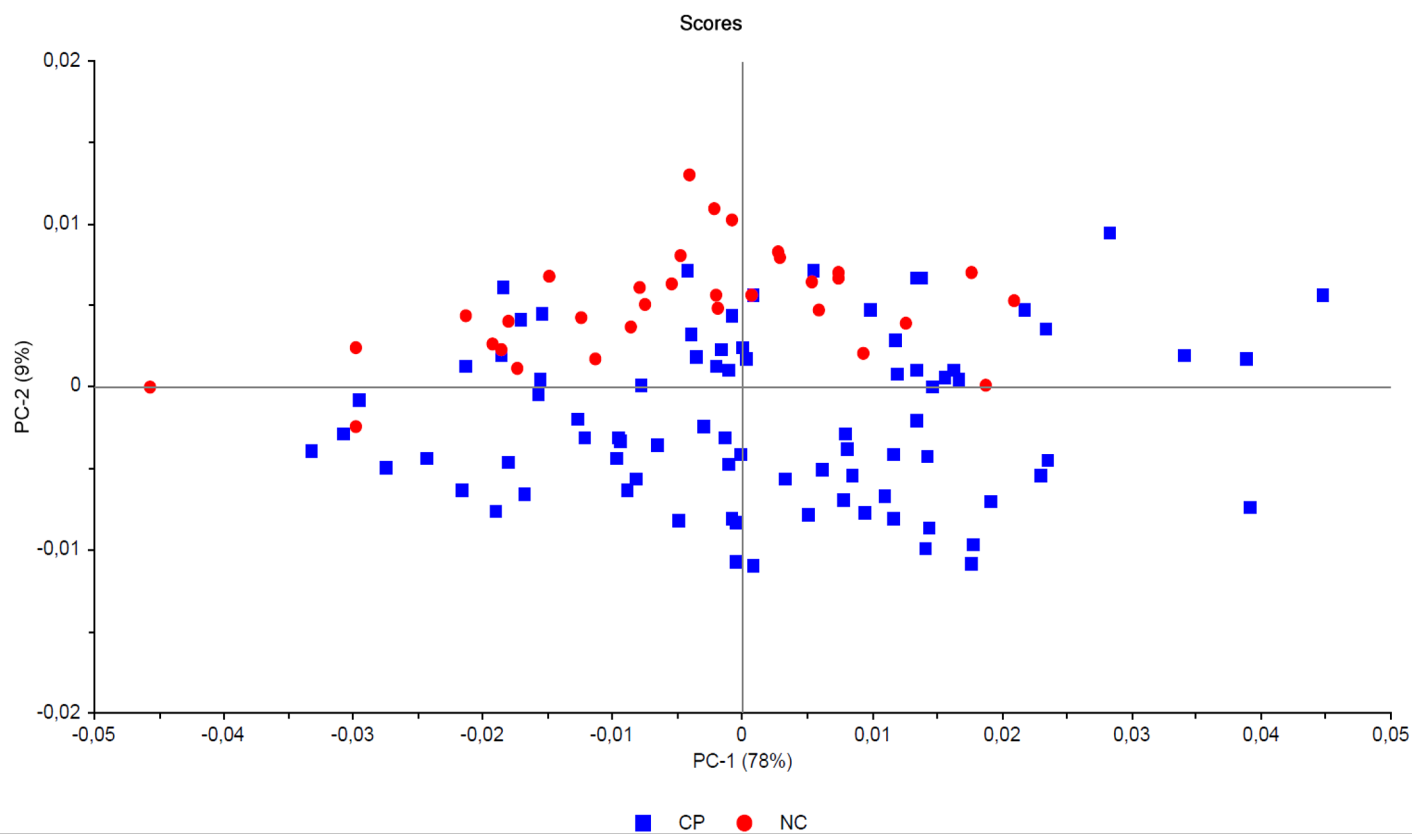

B

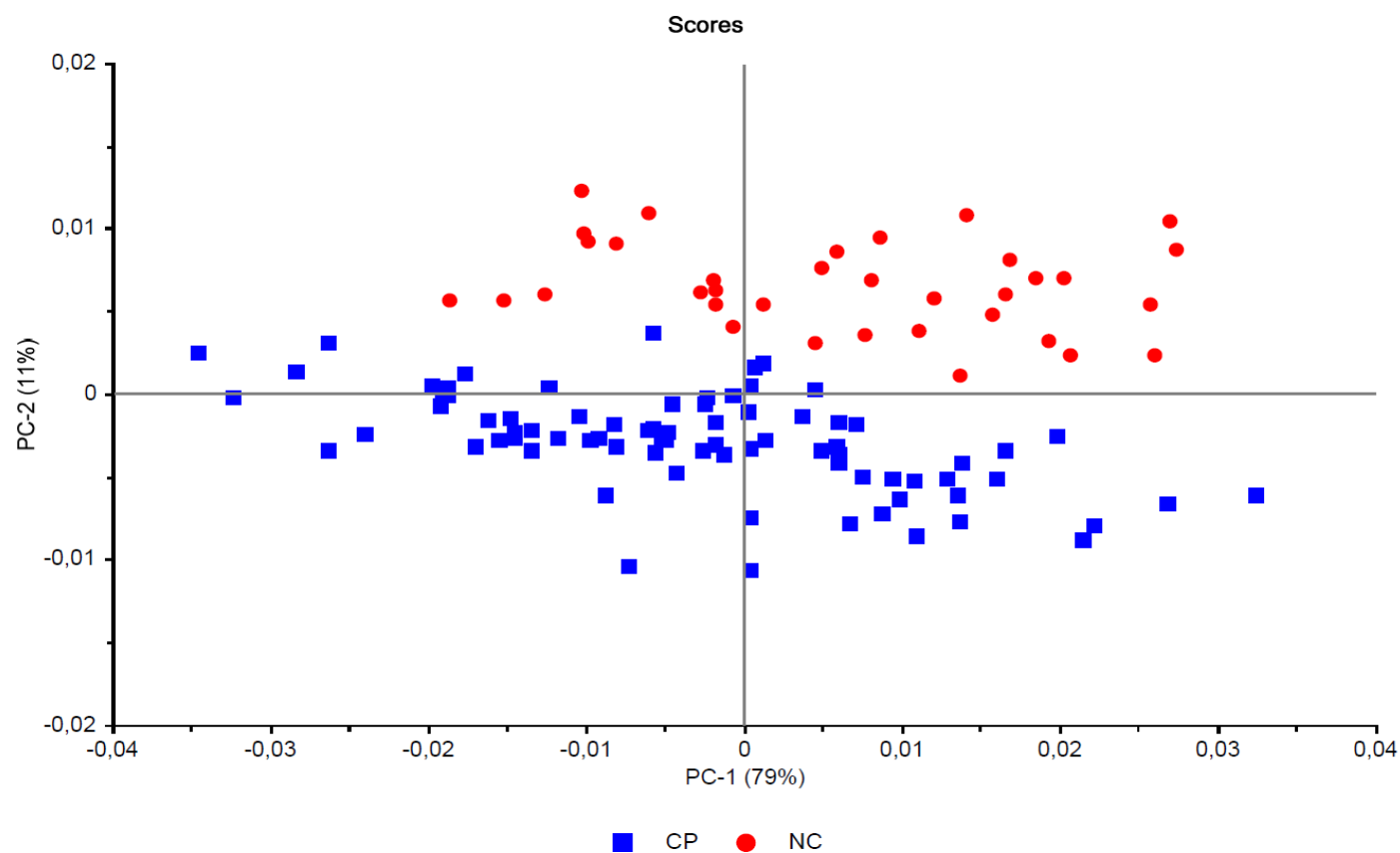

Supplement: Supplementary file 1 — Additional file 1: Figure S1. Principal component analysis (PCA) of non-copulated Lutzomyia longipalpis females at different ages. (a) One (blue squares), 3 (brown triangles), 8 (blue stars), 10 (red circles), 15 (green triangles), and 17 (light blue diamonds) days old; (b) 1 (blue squares), 8 (red circles), and 15 (green triangles) days old; (c) < 8 (blue squares) or ≥ 8 (red circles) days old. Figure S2. Loadings of PCA [principal component 1 (PC – 1)] of non-copulated Lutzomyia longipalpis females at different ages: (a) 1-8-15 days old and (b) < 8 or ≥ 8 days old. Figure S3. Loadings of PCA [principal component 1 (PC – 1)] of non-copulated Lutzomyia longipalpis males at different ages: (a) 1-3-8-10-15-17 days old, (b) 1 8 15 days old, and (c) < 8 or ≥ 8 days old. Figure S4. Principal component analysis (PCA) of non-copulated Lutzomyia longipalpis males at different ages. (a) One (blue squares), 3 (brown triangles), 8 (gray stars), 10 (red circles), 15 (green triangles), and 17 (light blue diamonds) days old; (b) 1 (blue squares), 8 (red circles), and 15 (green triangles) days old; (c) < 8 (blue squares) or ≥ 8 (red circles) days old. Figure S5. Loadings of PCA [principal component 1 (PC – 1)] of copulated Lutzomyia longipalpis females at different ages: (a) 1-8-15 days old and (b) < 8 or > 8 days old, zoomed in. Figure S6. Loadings of PCA (principal component 1 (PC – 1)) of copulated Lutzomyia longipalpis males at different ages: (a) 1-8-15 days old and (b) < 8 or > 8 days old. Figure S7. Principal component analysis (PCA) of copulated Lutzomyia longipalpis at different ages. (a) One (blue squares), 8 (green triangles), and 15 (red circles) days old; (b) < 8 (blue triangles) or > 8 (red circles) days old. Figure S8. Principal component analysis (PCA) of copulated Lutzomyia longipalpis males at different ages. (a) One (blue squares), 8(green triangles), and 15 (red circles) days old; (b) < 8 (blue squares) or > 8 (red circles) days old. Figure S9. Principal compon [file 13071_2023_6097_MOESM1_ESM.zip › Supple fig/Supplementary Figure 9.pdf]
